# *Swertia cincta* Burkill alleviates LPS/D-GalN-induced acute liver failure by modulating apoptosis and oxidative stress signaling pathways

**DOI:** 10.18632/aging.204848

**Published:** 2023-06-27

**Authors:** Xinyan Wu, Xiaomei Zheng, Qiqi Wen, Yang Zhang, Huaqiao Tang, Ling Zhao, Fei Shi, Yinglun Li, Zhongqiong Yin, Yuanfeng Zou, Xu Song, Lixia Li, Xinghong Zhao, Gang Ye

**Affiliations:** 1 College of Veterinary Medicine, Sichuan Agricultural University, Wenjiang, Chengdu, China

**Keywords:** acute liver failure, network pharmacology, apoptosis, *Swertia cincta* Burkill extract, oxidative stress

## Abstract

*Swertia cincta* Burkill is widely distributed along the southwestern region of China. It is known as “Dida” in Tibetan and “Qingyedan” in Chinese medicine. It was used in folk medicine to treat hepatitis and other liver diseases.

To understand how *Swertia cincta* Burkill extract (ESC) protects against acute liver failure (ALF), firstly, the active ingredients of ESC were identified using liquid chromatography-mass spectrometry (LC-MS), and further screening. Next, network pharmacology analyses were performed to identify the core targets of ESC against ALF and further determine the potential mechanisms. Finally, *in vivo* experiments as well as *in vitro* experiments were conducted for further validation. The results revealed that 72 potential targets of ESC were identified using target prediction. The core targets were ALB, ERBB2, AKT1, MMP9, EGFR, PTPRC, MTOR, ESR1, VEGFA, and HIF1A. Next, KEGG pathway analysis showed that EGFR and PI3K-AKT signaling pathways could have been involved in ESC against ALF. ESC exhibits hepatic protective functions via anti-inflammatory, antioxidant, and anti-apoptotic effects. Therefore, the EGFR-ERK, PI3K-AKT, and NRF2/HO-1 signaling pathways could participate in the therapeutic effects of ESC on ALF.

## INTRODUCTION

ALF is a life-threatening disease frequently exhibiting a fulminant progression [1]. ALF patients have a high mortality rate, and approximately 30% undergo liver transplantation [2]. Despite improving survival rates, ALF remains a devastating disease having a high mortality rate. Hepatitis virus, ischemia-reperfusion injury of the liver, drug overdose, and toxic substances are some of the most common causes of ALF [3]. Only limited pharmacological treatment options are available for ALF, including corticosteroids, antiviral drugs, and immunosuppressants. However, the long-term options for using these drugs can cause severe side effects and liver damage [4]. Therefore, it is necessary to identify new agents to treat ALF effectively.

Recently, traditional Chinese medicine (TCM) has become more popular due to its multi-ingredient, multi-link, and multi-target benefits [5]. The global distribution of 170 species of Swertia is found in the Gentianaceae family, comprising 87 genera [6], most of which have been widely utilized by TCM for liver disease.

*Swertia cincta* Burkill is an annual herb from Swertia of Gentianaceae that can clear the liver and gallbladder’s dampness and heat [7]. It is mostly available in Southwest China, such as Sichuan and Tibet. Meanwhile, as the Dida in Tibetan medicine, *Swertia cincta* Burkill is utilized as folk medicine by Hani and Tibetan to treat hepatitis, icteric hepatitis, and other liver diseases [8]. Pharmacology studies have revealed that ESC possesses hepatoprotection against CCl4-induced liver injury and HBV [9, 10]. In addition, reports have revealed that the ESC against α-naphthylisothiocyanate-induced cholestasis in rats regulates the hepatic transporter and metabolic enzyme expression [11]. However, in the Chinese Pharmacopoeia, only *Swertia mileensis*, a plant from the same genus, has been included as an anti-hepatitis drug. Extensive research has depicted that the two species have similar phytochemical ingredients, such as swertiamarin, gentiopicroside, sweroside, and mangiferin [7, 12]. Altogether, *Swertia cincta* Burkill could be a promising hepatoprotective agent, warranting further research into its mechanisms.

Network pharmacology is an emerging, interdisciplinary, and cutting-edge discipline, emphasizing the elucidation of disease and drug mechanisms from a holistic perspective. [13]. Previous studies have shown that Chinese herbal medicines (CHM) contain a complex composition, and their pharmacological actions may be due to their synergistic effects on multiple ingredients [14]. Fortunately, since network pharmacology emerged, it has become a powerful tool for CHM. Therefore, network pharmacology had been used by researchers to investigate drug targets and efficacy in CHM. In recent years, there also had been more and more studies to investigate the mechanism of CHM in the treatment of ALF by network pharmacology [15].

In this study, using the network pharmacology strategy, the main targets and signaling pathways of the protective effect of ESC on ALF were predicted and verified by *in vivo* experiments as well as *in vitro* experiments, which is expected to provide a scientific basis for the development and pharmaceutical value of ESC.

## RESULTS

### The identification of components in ESC

**Figure 1:**
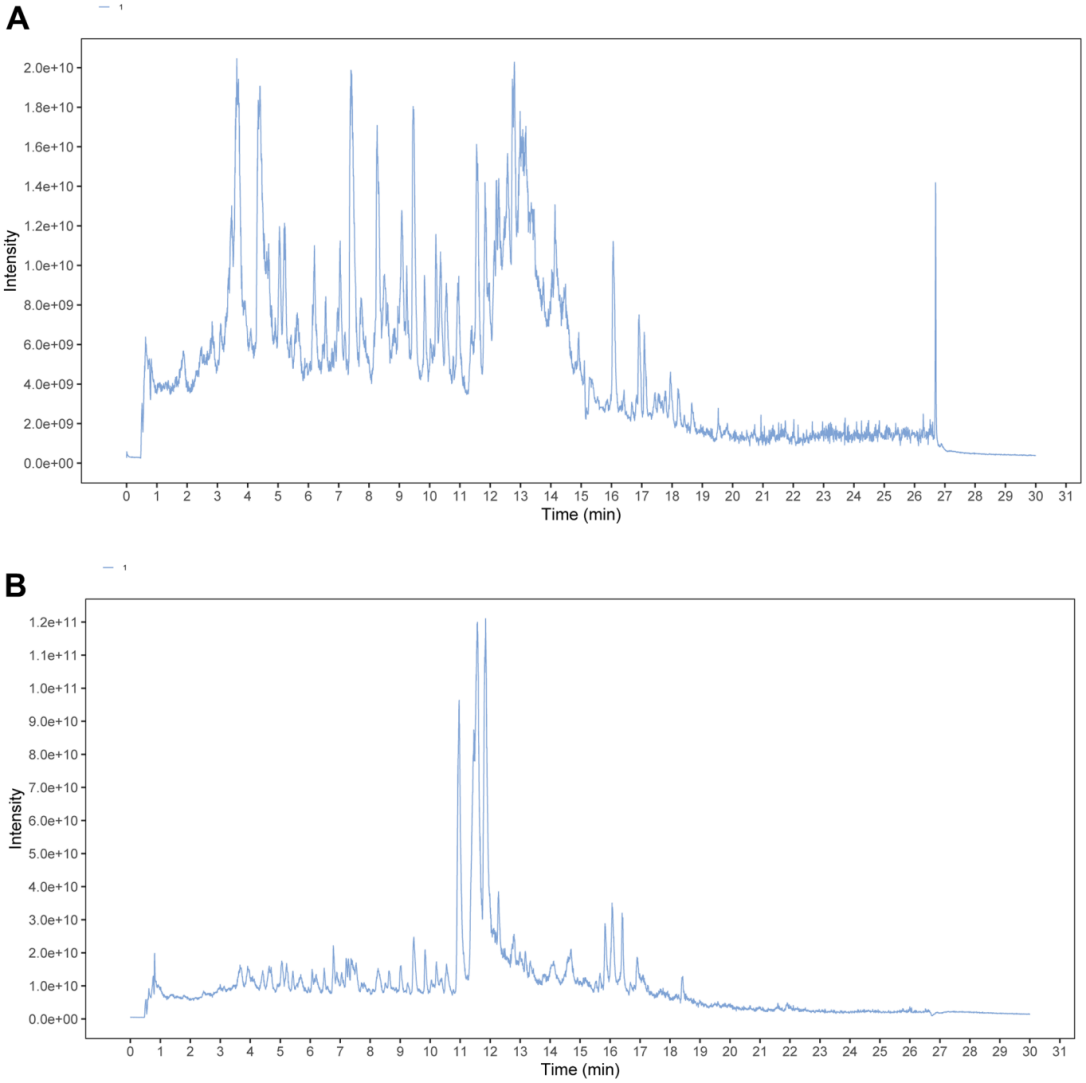
**TIC chromatograms of ESC.** (**A**) Negative mode. (**B**) Positive mode.

As shown in Figure 1, a total of 41 compounds were detected from ESC, including flavonoids, alkaloids, iridoids, miscellaneous, and terpenoids. The detailed information is listed in Supplementary Table 1. All the compounds show a deviation between the theoretical and measured m/z of fewer than 5 ppm.

### Screening for active components of ESC

**Figure 2:**
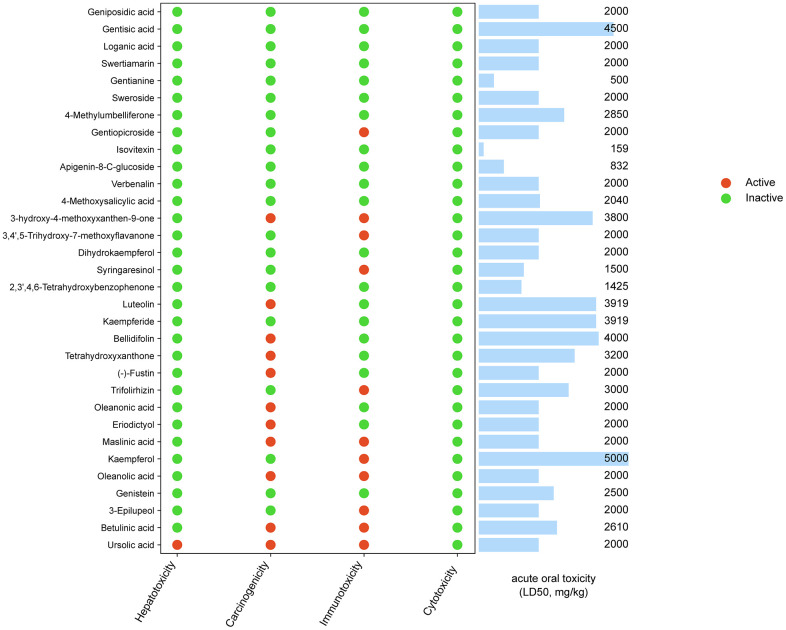
Toxicity of ESC main components.

We set a condition to screen the components of ESC. First, compounds obeying at least three criteria were considered to adhere to Lipinski Rule. Second, the Bioavailability Score is ≥0.55. The compound was eliminated from the candidates when the above two conditions were violated simultaneously. This screening yielded a total of 32 candidate compounds (Supplementary Table 6). The toxicity of candidate compounds was predicted with the ProTox-II server, and the results have been summarized in Figure 2. The results demonstrated that most candidate compounds (31/32, 97%) showed no measurable hepatotoxicity except ursolic acid.

### Identification of candidate targets in ESC for ALF treatment

**Figure 3:**
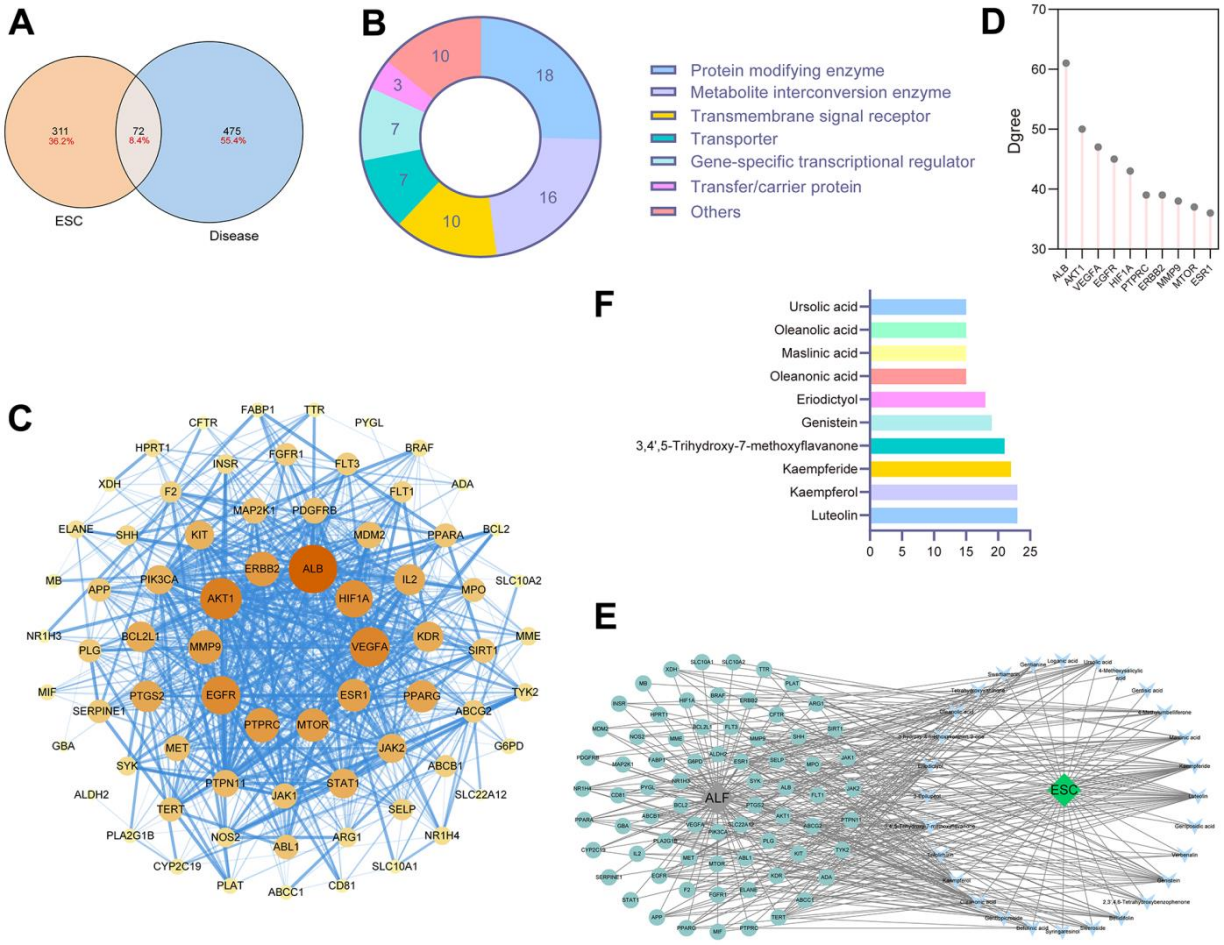
**Network pharmacology analysis for components in ESC.** (**A**) The intersection of ESC targets and ALF targets. (**B**) The protein classification of targets of ESC against ALF. (**C**) PPI networks of ESC against ALF-related targets. (**D**) The degree score of core targets. (**E**) ESC-ingredient-target network. (**F**) Top 10 key components in ESC.

383 targets were obtained from SwissTarget, Sea, and Herb to determine the ESC-associated targets. After searching ‘acute liver failure’ and ‘acute liver injury’ in DrugBank, Genecard, TTD, OMIM, and PharmGKB databases, 547 disease targets were associated with ALF. Then, we took the intersection of the two prediction results to secure high-confidence ESC against ALF targets, and a total of 72 potential targets were retrieved (Figure 3A). These targets are detailed in Supplementary Table 2. Next, 72 identified proteins were categorized into 14 protein classes based on the PANTHER Classification System (Figure 3B). Protein modifying enzyme (25%), metabolite interconversion enzyme (22.5%), transmembrane signal receptor (14%), transporter (10%), and gene-specific transcriptional regulator (10%) were the top 5 protein classes. Non-receptor serine/threonine protein kinase was the primary type in protein-modifying enzymes, such as AKT1, MTOR, MAP2K1, and BARF. Three protein types mainly exist among the metabolite interconversion enzyme: oxidoreductase, dehydrogenase, and oxygenase.

### PPI network and screening of core targets

We constructed candidate targets involved in PPI network by using the STRING database, as shown in Figure 3C. Ranking by degree, we selected the top 10 predicted targets in the PPI network as the network core target in ESC for ALF treatment. In order of degree scores from high to low were as follows ALB, ERBB2, AKT1, MMP9, EGFR, PTPRC, MTOR, ESR1, VEGFA, and HIF1A (Figure 3D). The result suggested these core targets could be critical in the therapeutic effects of ESC for ALF. A network of ingredient targets was constructed for each of the 72 intersection targets to provide a general overview of interactions between herbs and ingredients (Figure 3E). The number of associated targets was used to rank each bioactive ingredient. The top 6 compounds were: luteolin, kaempferol, kaempferide, 3,4’,5-Trihydroxy-7-methoxyflavanone, genistein, and eriodictyol (Figure 3F). This could hint at the positive role of these ingredients in ESC for ALF treatment.

### GO function and KEGG pathway enrichment analysis

**Figure 4:**
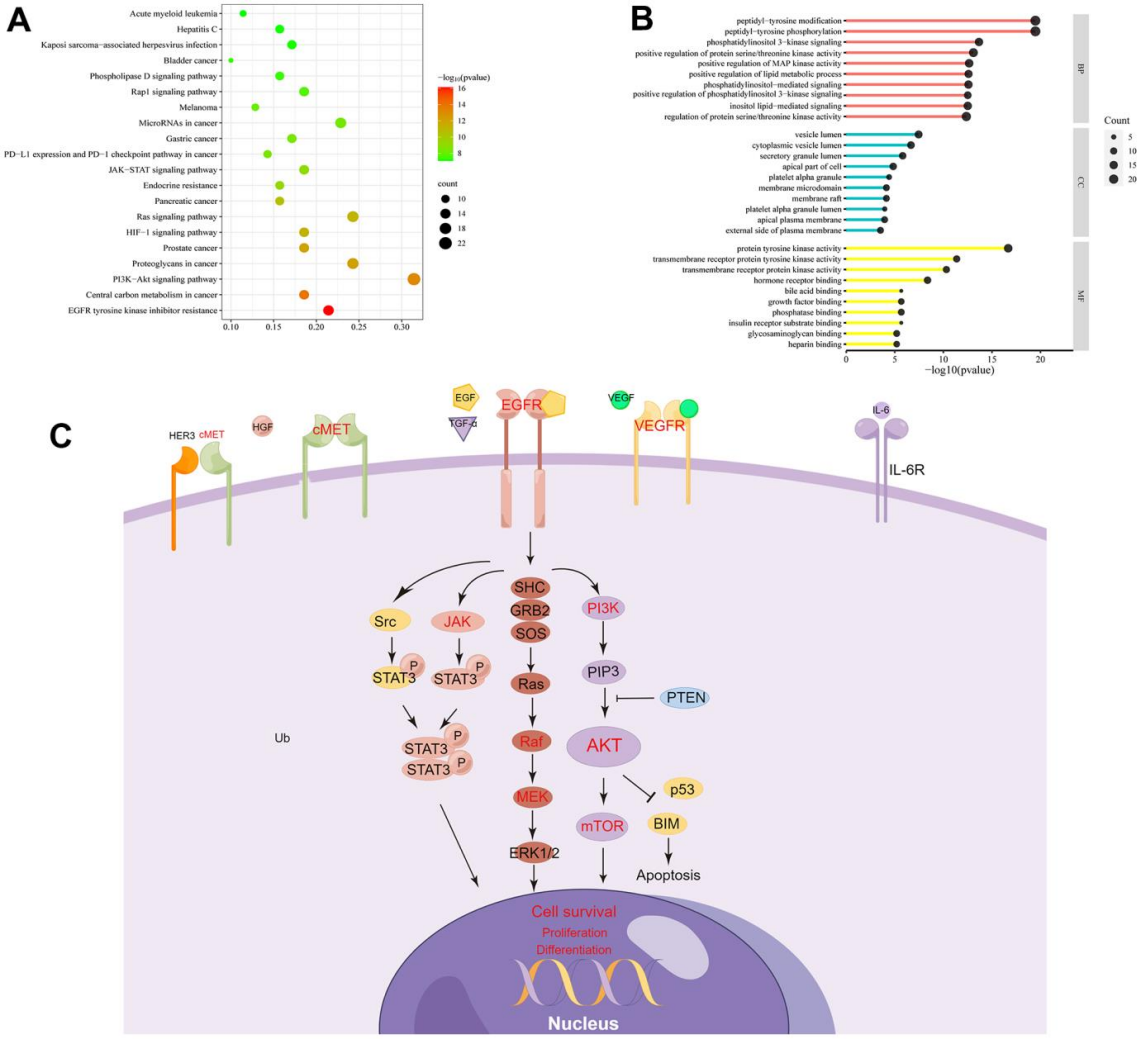
**GO and KEGG enrichment analysis.** (**A**) The top 20 pathways enriched in KEGG. (**B**) GO shows the Top 10 of BP, CC, and MF. (**C**) Genes related to EGFR signaling pathways are represented in a mechanistic diagram.

In the GO enrichment analysis, 3 aspects were included: biological process (BP), cellular component (CC), and molecular function (MF). These validate that ESC was enriched in various BP terms, including “peptidyl-tyrosine phosphorylation” (GO: 0018108), “peptidyl-tyrosine modification” (GO: 0018212), and “phosphatidylinositol 3-kinase signaling” (GO: 0014065). The term CC was enriched in the vesicles like “vesicle lumen” (GO: 0031983), “secretory granule lumen” (GO: 0034774), and “cytoplasmic vesicle lumen” (GO: 0060205). The MF term was primarily responsive, including “protein tyrosine kinase activity” (GO: 0004713) and other “transmembrane receptor protein tyrosine kinase activity” (GO: 0004714). These are briefly listed in Supplementary Table 3 and visualized by SRplot (Figure 4B).

After KEGG analysis, the top 20 enriched KEGG pathways were reported (Figure 4A). The detailed information is represented in Supplementary Table 4. “EGFR tyrosine kinase inhibitor resistance” and “PI3K-Akt signaling pathway” were two critical pathways associated with ESC against ALF. In the following study, we further examined the key signaling molecules in these two downstream pathways (Figure 4C).

### Expression of key genes in ALF liver tissues in GEO database

**Figure 5:**
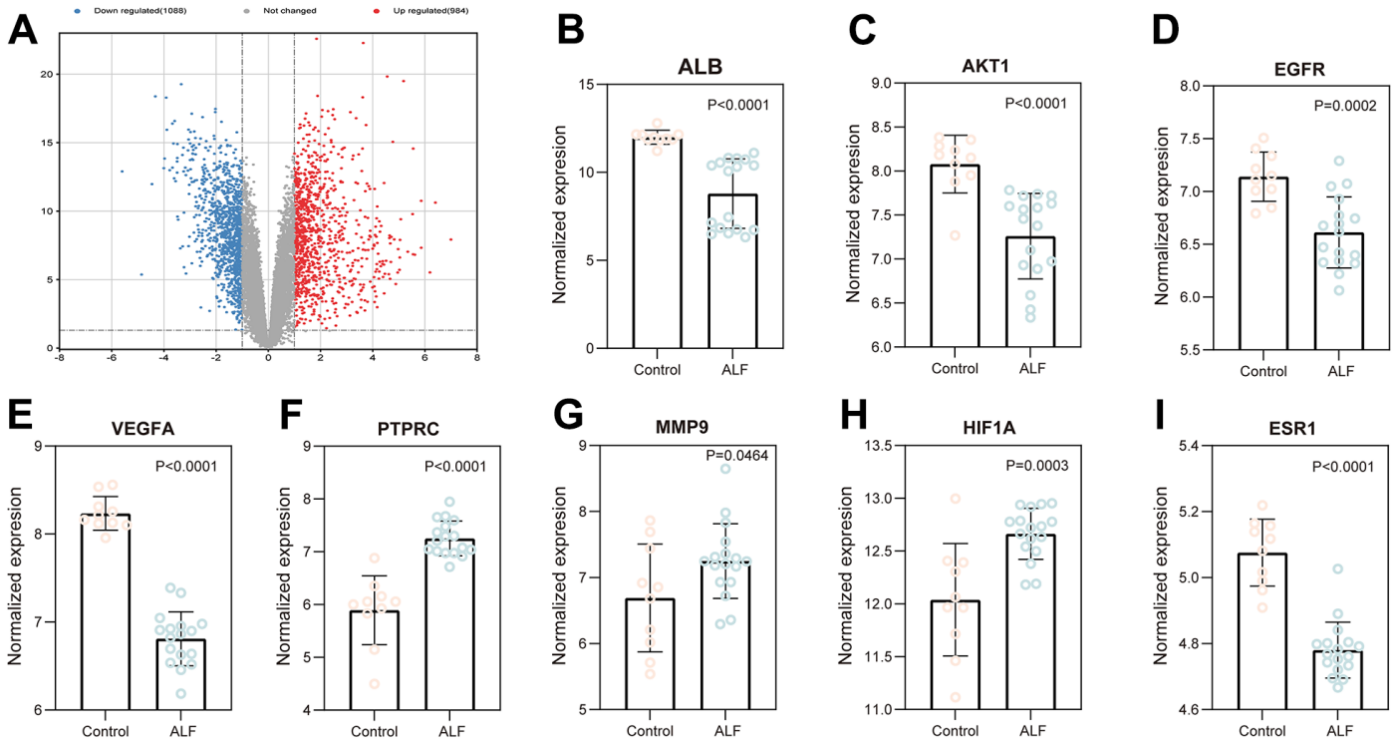
**Validation of core targets using the data from the GEO database.** (**A**) Volcano map analysis of ALF. The expression of ALB (**B**), AKT1 (**C**), EGFR (**D**), VGEFA (**E**) PTPRC (**F**), MMP9 (**G**), HIF1A (**H**), and ESR1 (**I**).

GSE datasets were selected from GEO (GSE38941) to verify these core targets. The volcano plot revealed that 1088 downregulated and 984 upregulated genes were found in the ALF group. The cut-off criteria were |log 2 (FC)| ≥ 2.0, p-value ≤ 0.05, and FDR ≤ 0.05 (Figure 5A). In addition, the expression of 10 key targets in liver tissues were compared between ALF and control groups. The livers of patients revealed a significant expression of eight of ten target genes than those of control livers (P < 0.05). In the ALF group, PTPRC, MMP9, and HIF1A were significantly over expressed than in the control group. In contrast, EGFR, AKT1, ESR1, VEGFA, and ALB expressions had been reversed (Figure 5B–5I). The expressions indicated that the key targets were associated with the ALF process. Furthermore, gene expression could be regulated by ESC to ameliorate ALF.

### Molecular docking analysis

**Figure 6:**
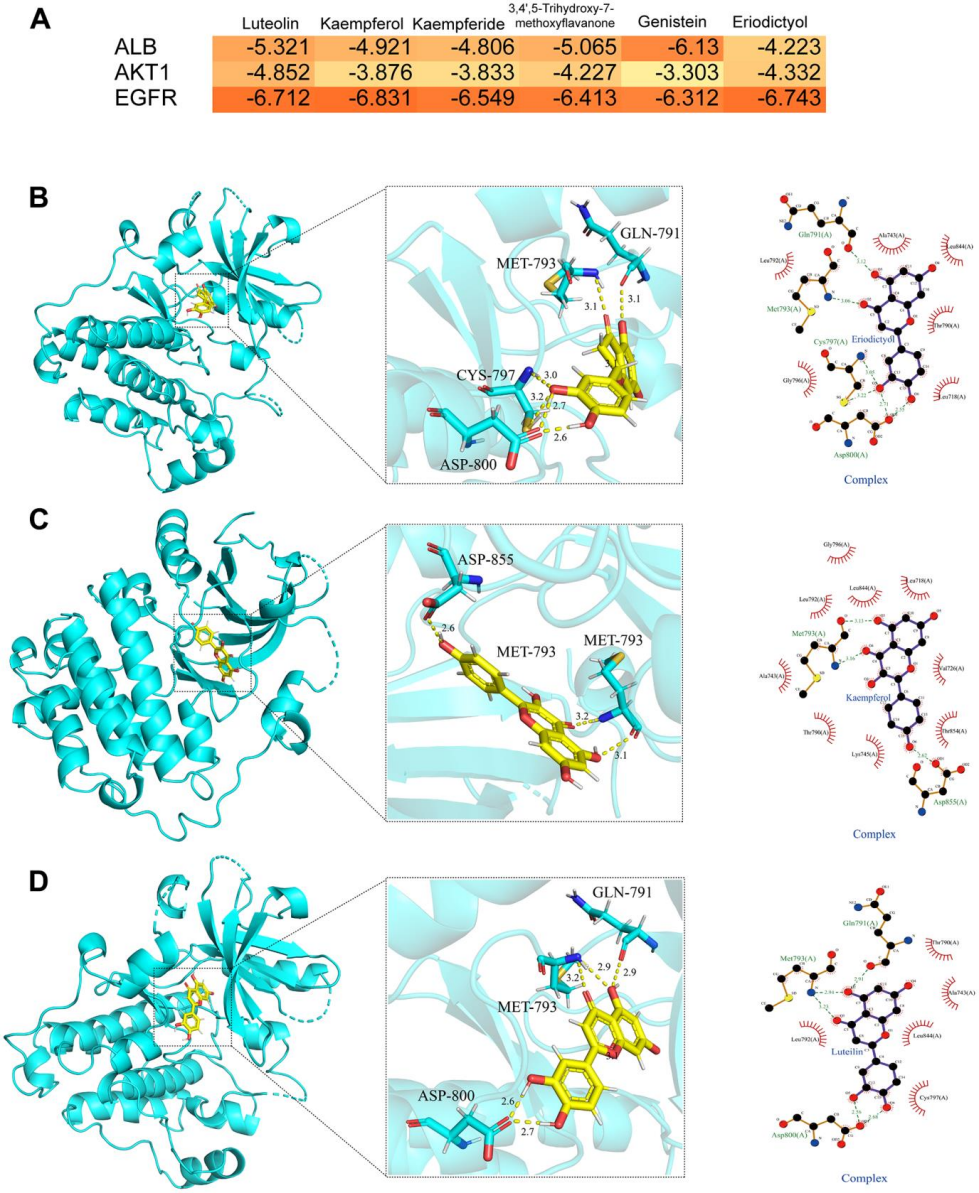
**Molecular docking analysis.** (**A**) The heat map of molecular docking scores (kcal/mol). (**B**) The binding modes of eriodictyol-EGFR complex (2D and 3D images). (**C**) The binding modes of kaempferol-EGFR complex (2D and 3D images). (**D**) The binding modes of luteolin-EGFR complex (2D and 3D images). Results of 3 independent experiments were described above.

We evaluated the binding between the key targets (ALB, EGFR, and AKT1) and the corresponding bioactive compounds (luteolin, kaempferol, kaempferide, 3,4’,5-Trihydroxy-7-methoxyflavanone, genistein, eriodictyol) using molecular docking based on the PPI topology analysis and GEO dataset verification. The hot map indicated the binding results depending on the docking scores (Figure 6A). Target proteins exhibited good binding energies in most cases. The associated compound with the lowest docking score for each target (luteolin-EGFR, eriodictyol-EGFR, and kaempferol-EGFR) was analyzed using receptor-ligand interaction, including binding site and distance (Figure 6B–6D).

### MD simulation to explore the interaction of the key ingredients for ESC on EGFR

**Figure 7:**
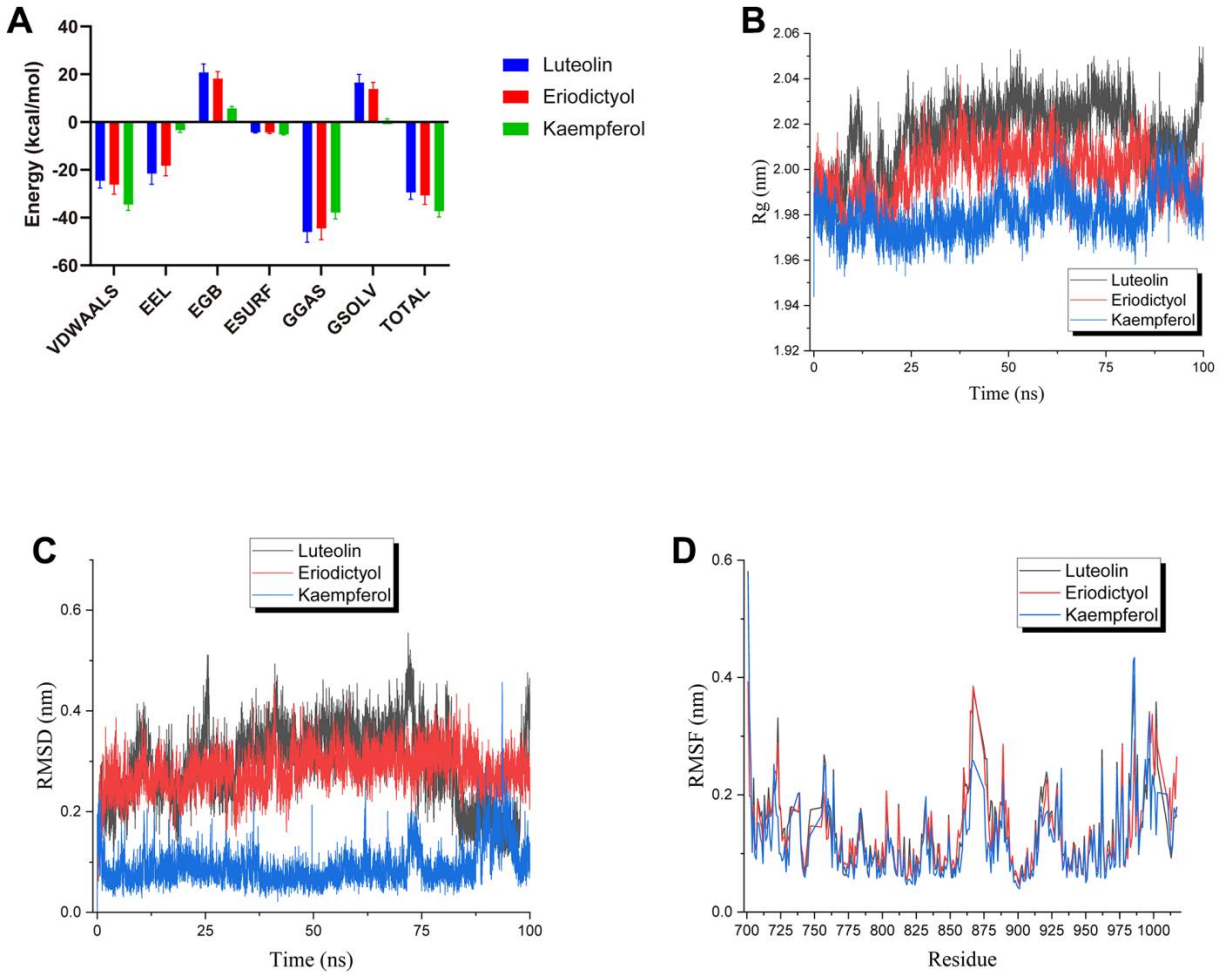
**Molecular dynamic simulations.** (**A**) The MM-PBSA module determined the total binding energy and the energy components. (**B**) The radius of gyration obtained in MD analysis. (**C**) Complex RMSD. (**D**) Complex RMSF.

The root means square deviation (RMSD) indicates structural stability. GROMACS g_rmsd tool was used to determine RMSD. Excluding the initialization steps, the starting structure of each simulation is the reference structure. The lower the RMSD, the more stable the protein complex. It can be observed from Figure 7C that luteolin, eriodictyol, and kaempferol within 100ns of the simulation were constant and low for the entire duration of the experiment. This revealed that the ligand was bound to the receptor, and the complex became stable. In particular, the RMSD value of kaempferol showed a very low RMSD (< 0.2 nm), implying the stable binding of kaempferol.

Root-mean-square fluctuations (RMSF) provide direct insight into the structural fluctuation and flexibility of proteins. The larger its value is, the stronger this protein residue interacts with small molecules will be. This residue is a critical amino acid for interacting with small molecule ligands. Based on Figure 7D, the distributions of RMSF values for luteolin, eriodictyol, and kaempferol were consistent. The RMSF fluctuation values of Res 861-Res 867 in kaempferol were smaller than the other two ligands. In contrast, the RMSF fluctuation values of Res 718-Res 719 and Res 984-Res 986 were higher than the other two ligands during the simulation. Thus, it hinted that the binding mode and critical residues of kaempferol could be different from the other two ligands. Overall, the RMSF fluctuation values of all three ligands had a low level during the simulation. This suggested that the complexes were stable.

The Radius of gyration (Rg) was analyzed to decipher the changes in the compactness of the protein. From Figure 7B, the Rg of proteins were all less than 2.1 nm, indicating a more stable, tighter complex formation during the simulation. Notably, kaempferol had a smaller Rg than the other ligands, depicting a stable complex with the protein.

Finally, the molecular mechanics Poisson-Boltzmann surface area calculation was utilized to validate the observations from the molecular docking method. Four energy terms contributed to the total binding energy of each ligand-protein complex: van der Waals, electrostatic, polar solvation, and non-polar solvation. The results demonstrated that van der Waals was the predominant force underlying the binding of the compounds to EGFR. Moreover, luteolin and eriodictyol had partial electrostatic interactions. In contrast, kaempferol had a significantly stronger interaction with the protein than luteolin and eriodictyol. Therefore, the overall binding free energy was higher than that of luteolin and eriodictyol.

### ESC protects against LPS/D-GalN-induced acute liver failure *in vivo*

**Figure 8:**
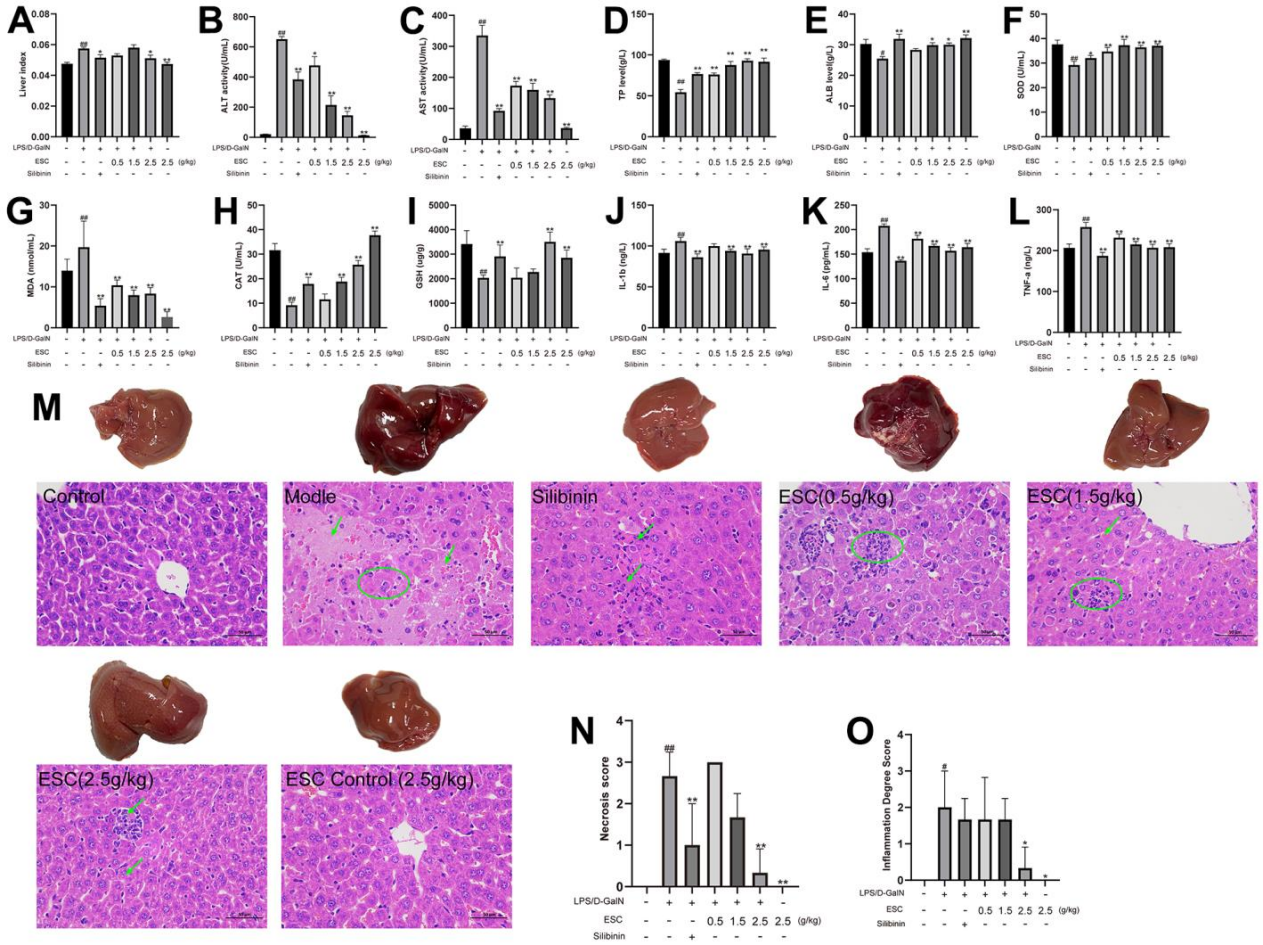
**ESC alleviated acute liver failure within an *in vivo* model.** (**A**) Liver index; (**B**) serum ALT activity, (**C**) AST activity, (**D**) TP levels, (**E**) ALB levels, (**F**) SOD levels, (**G**) MDA levels, (**H**) CAT levels, (**I**) GSH levels, (**J**) IL1-β levels, (**K**) IL-6 levels, and (**L**) TNF-α levels; (**M**) The histopathology of mice liver (HE 400×); (**N**) Necrosis score; (**O**) inflammation degree score. Results of 6 independent experiments were described above, of which the significant ones were recorded as *p < 0.05, **p < 0.01 and #p < 0.05, ##p < 0.01, respectively, for the model group and the control group.

After treatment using LPS/D-GalN, the ALT, AST, MDA, IL-1β, IL-6, and TNF-α levels in the mice increased significantly. However, ALB, TP, SOD, CAT, and GSH decreased considerably. Additionally, the liver index of the mice in the model group increased significantly compared with the control group. Conversely, different concentrations of ESC pre-treatment significantly decreased the LPS/D-GalN-induced phenomenon (Figure 8A–8L).

**Figure 9:**
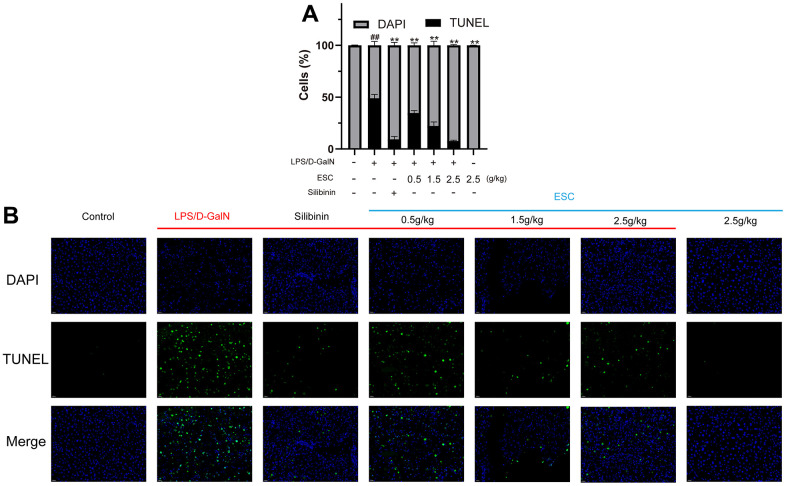
**TUNEL histology for mice.** (**A**) The calculated percent TUNEL-positives cells. (**B**) The representative TUNEL stain images (400×). Results of 6 independent experiments were described above, of which the significant ones were recorded as *p < 0.05, **p < 0.01 and #p < 0.05, ##p < 0.01, respectively, for the model group and the control group.

The histological assessment exhibited a similar trend, and the gross appearance demonstrated the hepatoprotective effects of ESC. In normal liver tissue, HE staining revealed neatly arranged hepatocytes without any infiltration of inflammatory cells. There was the destruction of liver lobules, hyperplasia, inflammatory cell infiltration in the model group, necrosis, and blood extravasation. The number and area of inflammatory cell infiltration and necrosis of hepatocytes were significantly reduced in all the high-dose ESC-treated groups compared with the model group (Figure 8M–8O). Additionally, the ALF model group had higher TUNEL positivity rates than the control group based on the TUNEL staining results. However, ESC decreased TUNEL-positive staining at ALF (Figure 9A, 9B).

### ESC protects against LPS/GalN-induced acute liver failure *in vitro*

**Figure 10:**
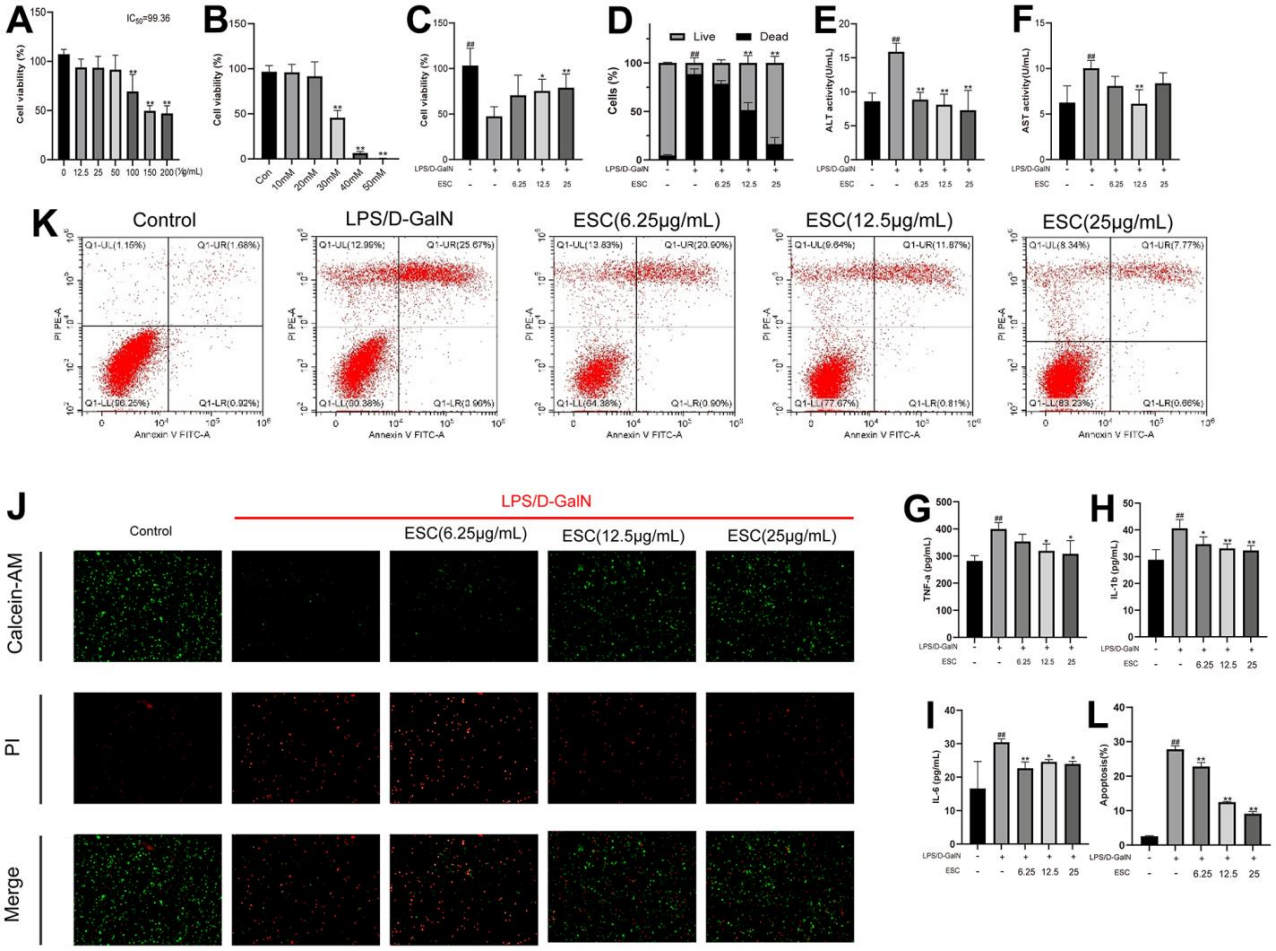
ESC alleviated acute liver failure *in vitro* (**A**) HepG2 cells were treated with 0-200 μg/mL ESC for 24 h. (**B**) HepG2 cells were treated with 0-50 mM D-GalN for 24 h. (**C**) HepG2 cells were exposed to 30mM D-GalN and 1 μg/mL LPS. Then, they were treated with 6.25, 12.5, and 25 μg/mL ESC medicated serum for 24 h or with blank serum. (**D**) Cell death percentage was determined by live/dead assay using fluorescent probe calcein-AM/PI. (**E**) The ALT, (**F**) AST, (**G**) TNF-α, (**H**) IL1-β, (**I**) IL-6 levels in the culture supernatant of HepG2 cells. (**J**) The calcein-AM/PI staining (100×). (**K**) The apoptotic status was measured through flow cytometry. (**L**) Percentages of apoptotic cells. Results of 3 independent experiments were described above, of which the significant ones were recorded as *p < 0.05, **p < 0.01 and #p < 0.05, ##p < 0.01, respectively, for the model group and the control group.

The effect of ESC on the viability of HepG2 was determined using CCK-8 assay to study the possible cytoprotective effects of ESC on HepG2 cells injured by LPS/D-GalN. As illustrated in Figure 10A, 10B, it showed significant cytotoxic activity toward these HepG2 cells at 30 mM and 100 ug when cells were incubated for 24 h at increasing concentrations (10-50 mM) or (12.5-200 ug/mL) of D-GalN or ESC. Considering the results mentioned above, we selected 30 mM D-GalN for modeling. Our results also revealed that after exposure to ESC for 24 h, the half-maximal inhibitory concentration (IC50) of HepG2 cells was 99.36 μg/mL. Thus, our results selected 0-25 μg/mL ESC for the next test. The following experiments further explore the effect of ESC treatment at gradient concentrations (6.25, 12.5, 25 ug/mL) in the LPS/D-GalN-induced HepG2 cell model. Our results revealed that the ESC groups (12.5 and 25 ug/mL) significantly enhanced the survival rates of HepG2 cells more than the model groups (Figure 10C). Additionally, the calcein-AM/PI staining results were consistent with CCK-8, which showed that ESC could improve the survival rate of HepG2 cells (Figure 10D, 10J), indicating that ESC inhibits cell death triggered by LPS/D-GalN. Furthermore, the results of flow cytometry experiments showed that ESC was able to partially attenuate apoptosis induced by LPS/D-GalN in a dose-dependent manner (Figure 10K–10L).

The ALT and AST activities in cell supernatant were determined to examine the degree of hepatocyte damage. Figure 10E, 10F indicate that the model group represented higher cell supernatant ALT and AST levels (p < 0.01). However, ESC treatment reversed the increase in cell supernatant ALT and AST levels. Moreover, a similar trend was observed in TNF-α, IL-1β, and IL-6 (Figure 10G–10I).

**Figure 11:**
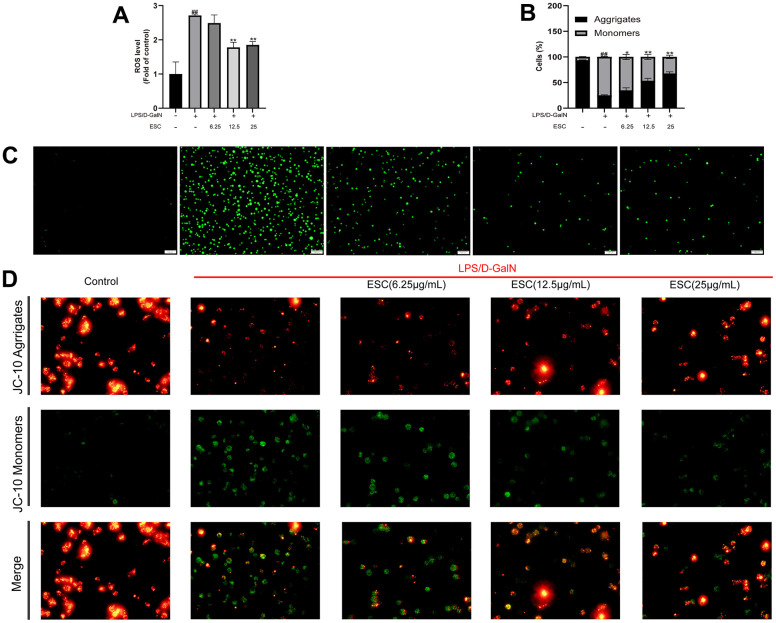
**The impact of ESC on ALF-mediated oxidative stress in HepG2 cells.** (**A**) ROS levels. (**B**) JC-10 red/green ratio. (**C**) Cellular ROS production was determined with the ROS probe (100×). (**D**) HepG2 cells were stained using JC-10. Results of 3 independent experiments were described above, of which the significant ones were recorded as *p < 0.05, **p < 0.01 and #p < 0.05, ##p < 0.01, respectively, for the model group and the control group.

Afterward, ROS levels were measured to determine whether ESC can decrease LPS/D-GalN-induced oxidative stress. As demonstrated in Figure 11C, intracellular ROS levels were significantly enhanced after 24 h of stimulation, hinting that LPS/D-GalN induces oxidative stress. Whereas intracellular ROS levels were significantly decreased by ESC (12.5 and 25 ug/mL) treatment (Figure 11A), the normal oxidation-reduction reaction state of cells was enhanced and restored to a certain extent.

Likewise, apoptosis, calcium homeostasis, ATP production, and ROS formation are controlled by mitochondria, producing cell energy [16]. Therefore, we examined the effect of ESC on MMP potential in ALF. As revealed in Figure 11D, the control group exhibited strong red fluorescence, indicating a high MMP. After being exposed to LPS/D-GalN for 24 h, the red/green cells reduced significantly, revealing that LPS/D-GalN promoted decreased MMP and impaired mitochondrial function (p < 0.01). MMP levels were significantly elevated or even reversed due to ESC treatment (Figure 11B).

**Figure 12:**
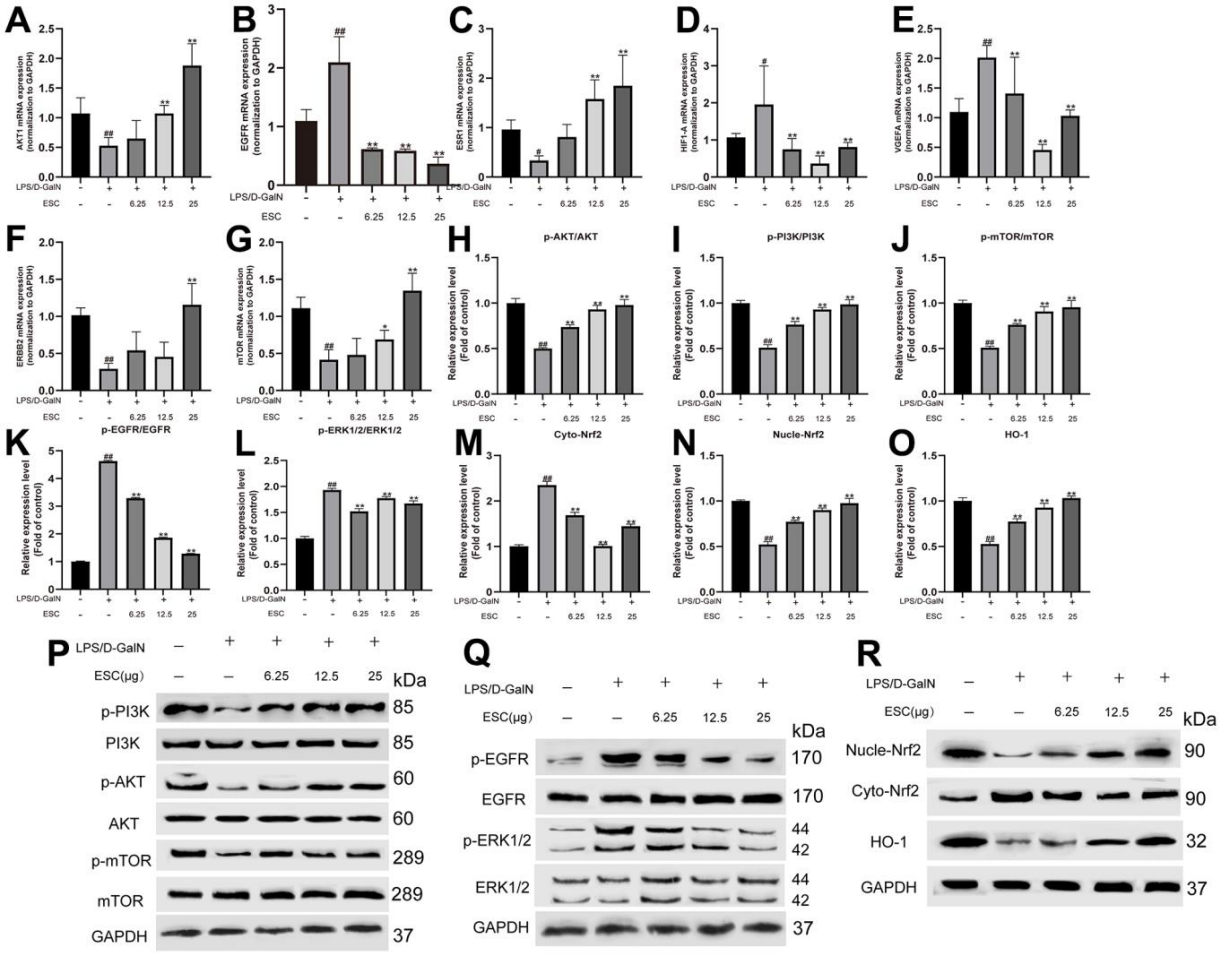
**ESC mechanism against ALF.** The mRNA expression of AKT1 (**A**), EGFR (**B**), ESR1 (**C**), HIF1A (**D**), VGEFA (**E**), ERBB2 (**F**), and mTOR (**G**). And the protein expression levels of p-AKT/AKT (**H**), p-PI3K/PI3K ratio (**I**), p-mTOR/mTOR ratio (**J**), p-EGFR/EGFR ratio (**K**), p-ERK1/2/ERK1/2 ratio (**L**), Cyto-Nrf2 (**M**), Nucle-Nrf2 (**N**), HO-1 (**O**), and (**P**–**R**) Western blot results. Results of 6 independent experiments were described above, of which the significant ones were recorded as *p < 0.05, **p < 0.01 and #p < 0.05, ##p < 0.01, respectively, for the model group and the control group.

Finally, the expression of the core targets for ESC against ALF was validated using qPCR. The results revealed that seven of the 10 core targets (AKT1, EGFR, ESR1, HIF1A, VEGFA, ERBB2, and MTOR.) significantly differed from the control group. However, compared with the model group, ESC pre-treatment significantly reversed these trends (Figure 12A–12G), contrary to VGEFA and EGFR expression in the GEO database. After this, the EGFR/ERK and PI3K/AKT/mTOR kinase cascade signaling pathways were analyzed based on the potential relationship between ESC and EGFR observed in molecular docking and molecular simulation analysis. Moreover, the Nrf2 protein was essential in oxidative stress and protects tissues and cells from oxidative stress. Furthermore, we studied the protein expression levels of Nrf2 and Nrf2-induced antioxidant protein HO-1. Figure 12I–12R detected that the p-AKT/AKT, p-PI3K/PI3K, p-mTOR/mTOR, Nucle-Nrf2, and HO-1 expression in the HepG2 cells sample were downregulated within the ALF model group, and the p-EGFR/EGFR, p-ERK1/2/ERK1/2 and cyto-Nrf2 were upregulated within the ALF model group. On the contrary, ESC significantly suppressed protein expression.

## DISCUSSION

ALF is characterized by rapid hepatocyte death due to many etiologies, such as systemic inflammatory response syndrome [17]. The development of efficient therapeutics for ALF is hindered by different unclear pathogenesis.

An LPS/D-GalN-induced animal model of ALF is widely used as the model of human liver failure to study mechanisms and potential therapeutic drugs in treating ALF [18]. D-GalN is a selective hepatotoxin and induces liver damage similar to viral hepatitis. In response to D-GalN “priming”, LPS-induced hepatic cell injury causes fulminant liver failure within 4–6 h of LPS/GalN administration [19]. In addition, LPS/D-GalN was applied to construct the *in vitro* ALF model [20, 21]. *Swertia cincta* Burkill had been widely utilized to treat various types of chronic and acute hepatitis and jaundice in Southwest China for hundreds of years. Meanwhile, *Swertia cincta* Burkill was anti-HBV [9] and anti-cholestasis [11]. However, *Swertia cincta* Burkill has rarely been reported in the study of ALF, and its relevant mechanism remains unclear.

At present, high-quality studies related to ESC is limited, and the vast majority of them focused on iridoid, flavonoid, and xanthones constituents, and that’s why our study focused on the analysis of the constituents identified in LC-MS. Therefore, studies with regard to the volatile constituents in ESC, detailed information [22] about which were displayed in Supplementary Table 5 for readers’ reference, should be carried out in the future. ESC was analyzed using UHPLC-QE Orbitrap/MS, total of 41 compounds in ESC were detected. Some of these compounds were reported to possess strong antioxidant and hepatoprotective effects by searching the literature, for instance, swertiamarin [23], gentiopicroside [24], kaempferol [25], and luteolin [26]. Therefore, further researches on the efficacy of the material basis of ESC against ALF are warranted.

We preliminarily identified the key targets and important pathways of ESC regulating ALF through network pharmacology to study the underlying mechanism. Among these core targets, EGFR was well known for its activity closely related to tumor growth, invasion, and metastasis. Meanwhile, EGFR expression is essential in maintaining cellular integrity and enabling intestinal epithelial cells to respond to injury [27]. Recently, studies indicated that EGFR could participate in ALF [28], alcohol-associated liver disease [29], and CCl4-induced liver fibrosis [30]. EGF and its tyrosine kinase receptor, EGFR, play a critical role in liver regeneration and transformation. The AKT kinases (AKT1, AKT2, and AKT3) are from the serine/threonine protein kinase family [31]. With over 85% homology, all three AKT isotypes share similar catalytic properties, can block apoptosis, and promote cell growth and metabolism. The hepatic deletion of AKT1 and AKT2 would induce liver injury and inflammation [32]. Simultaneously, AKT1 and EGFR are vital proteins in the EGFR/ERK signaling pathway and PI3K/AKT signaling pathway, respectively. Thus, we will focus on these two proteins in the future.

In addition, KEGG pathways and GO enrichment analysis were performed to explore the multi-dimensional pharmacological mechanism of ESC against ALF depending on the predicted genes. In the KEGG pathway analysis, EGFR tyrosine kinase inhibitor resistance (hsa01521), Central carbon metabolism in cancer (hsa05230), and PI3K/AKT signaling pathway (hsa04151) became the top three significantly enriched. Among these pathways, the central carbon metabolism in cancer was not closely associated with studying ESC against ALF. Moreover, EGFR tyrosine kinase inhibitor resistance was primarily concerned with the mechanisms of drug resistance in cancer and anticancer treatments. However, the MAPK/extracellular signal-regulated Kinases (ERK1/2) and the PI3K/AKT signaling pathways were the major downstream effectors of EGFR. Additionally, the predicted targets were primarily enriched in these two pathways. Currently, there are several reports on these signaling pathways in liver disease. Feng et al. [33] observed that matrine derivatives bind EGFR on HSC-T6 cells. Thus, it inhibited EGFR phosphorylation and its downstream protein kinase B, protecting hepatic parenchymal cells and enhancing hepatic functions. It has also been reported that the P2Y2 receptor regulates alcoholic liver inflammation by targeting the EGFR-ERK1/2 pathway and played a critical role in hepatocyte apoptosis [29]. Based on the above evidence, more attention is demanded in the EGFR/ERK1/2 and PI3K/AKT signaling pathways.

Depending on the above studies *in silico*, we speculated that ESC may convey hepatoprotective effects via its anti-oxidative and anti-apoptosis properties. Moreover, studies *in vitro* indicated that the anti-apoptotic effect of ESC might be exerted by controlling the PI3K/AKT and EGFR-ERK signaling pathways. Nrf2 is a crucial regulator of antioxidant stress responses transactivating a broad spectrum of enzymes involved in antioxidation, detoxification, cell survival, anti-inflammatory response, and more upon oxidative stress [34]. Furthermore, Nrf2 activation requires PI3K/AKT signaling pathways [35]. In the present study, the effects of ESC on ALF-induced oxidative stress were observed in both vitro and vivo. In addition, as previously reported by Fu et al. [36], α-mangostin could protect against LPS/D-GalN-induced ALF by upregulating the expressions of Nrf2 and HO-1 to induce antioxidant defense, which seemed to throw light on that Nrf2 was involved in LPS/D-GalN-induced ALF. Therefore, we suspected that ESC may account for the Nrf2-mediated antioxidant response stimulation.

Finally, our study identified several key components by molecular docking and MD (e.g., eriodictyol, maslinic acid) in ESC with an excellent protective effect against ALF. However, the effect of these components on liver disease has rarely been reported. Further investigations are required in future studies.

## CONCLUSIONS

Our study confirmed the beneficial and protective effect of ESC against ALF *in vivo* and *in vitro* and demonstrated that ESC might alleviate LPS/D-GalN-induced apoptosis in ALF by regulating the EGFR/ERK axis and its downstream mediators (PI3K/AKT), as well as reducing oxidative stress by altering Nrf2/HO-1 pathway and prevent mitochondrial damage. Therefore, we speculate that ESC could be an effective natural liver defender against ALF by boosting the anti-apoptosis and anti-oxidative effects, and this effect may be realized through EGFR/ERK, PI3K/AKT, and NRF2/HO-1 signaling pathways.

## MATERIALS AND METHODS

### Chemicals and reagents

Shanghai Yuan ye Bio-tech Co., Ltd. (Shanghai, China) supplied us with silibinin (≥98%, suspended in 0.5% carboxymethylcellulose sodium solution) and D-galactosamine (D-GalN). Lipopolysaccharide (LPS) (O55:B5), purchased from Sigma (Sigma, USA) coupled with D-GalN, was dissolved in saline.

The antibodies used (and their concentrations) were listed below: p-PI3K (CAT#4228, 1 : 1000, CST, USA), PI3K (CAT#4292, 1 : 1000), p-EGFR (CAT# ab40815, 1 : 1000), EGFR (CAT# ab52894, 1 : 1000), p-AKT (CAT# ab38449, 1 : 1000), AKT (CAT#4691, 1 : 1000), p-mTOR (CAT#5536, 1 : 1000), mTOR (CAT#2972, 1 : 1000), p-ERK1/2 (CAT#4370T, 1 : 1000, CST, USA), ERK1/2 (CAT#9102S, 1 : 1000), Nrf2 (CAT# ab137550, 1 : 1000), HO-1 (CAT# ab52947, 1 : 1000), GAPDH (CAT# 60004-1-Ig, 1 : 1000).

### Plant material

The whole plant of fresh *S. cincta* Burkill was obtained from the Lotus Pond Chinese herbal medicine market in Chengdu, Southwest China, in October 2020. The plant was identified by Prof. Lixia Li from Sichuan Agricultural University, Chengdu, China. The voucher specimen was preserved in the College of Veterinary Medicine, Sichuan Agricultural University, Chengdu, China (No. 20201018-1).

### Sample preparation

Firstly, 50 g of pulverized *S. cincta* Burkill (whole plant) were accurately weighed, and the sample was extracted twice with 75% (volume fraction) ethanol (w:v=1:20) through heating reflux twice, each time for 1h. Subsequently, the extract was filtered, concentrated with a vacuum rotary evaporator under reduced pressure, and lyophilized. Finally, 18.36 g ESC was obtained (2.72 g crude drugs per gram freeze-dried powder). 50 mg of them was taken into an EP tube, adding 1000μL extraction liquid (V methanol: V acetonitrile: V water = 2:2:1) and 20μL internal standard. Then, it’s homogenized in a ball mill at 45 Hz for 4 min and treated by ultrasound for 5min; three repetitions after, it was incubated for 1 h at -20° C and centrifuged at 12000 rpm at 4° C for 15 min. 200 μl supernatants were taken for the UHPLC-QE Orbitrap/MS analysis. Next, the remaining freeze-dried extract was prepared to powder with normal saline (for *in vivo*, the doses were equivalent to crude medicine amount when calculated according to the traditional dose for human (5–15 g/day)) or DMSO (for *in vitro*, and the concentration of DMSO is less than 0.1%) into high, medium, and low concentration suspensions or solution, respectively. The sample was stored at −20° C until used for subsequent experiments.

### LC-MS analysis

The following were the chromatographic conditions: UHPLC system (1290, Agilent Technologies), UPLC HSS T3 column (1.8μm, 2.1*100 mm, Waters), positive mode: mobile phase A was water with 0.1% formic acid, while mobile phase B was acetonitrile and negative mode: mobile phase A was water with 5 mM ammonium acetate, while mobile phase B was acetonitrile. Each sample was analyzed three times. The gradient elution was programmed as follows: 0 min, 1% B; 1 min, 1% B; 8 min, 99% B; 10 min, 99% B; 10.1 min, 1% B; 12 min, 1% B, which was delivered at 0.5 mL·min-1. Thermo Q exactive orbitrap mass spectrometer can collect primary and secondary mass spectrometry data under the control of XCalibur (version 4.0.27). Collision energy (NCE mode): 20eV, 40eV, 60eV, scanning rate: 7Hz. See Table 1 for detailed parameters.

**Table 1:** Mass spectrum parameters.

**QE**	**Positive**	**Negative**
Spray voltage (kV)	3.8 ESI+	3.1 ESI-
Capillary temperature (° C)	320	320
Sheath gas flow rate (Arb)	45	45
Aux gas flow rate (Arb)	15	15
mass range (m/z)	70-1000	70-1000
Full ms resolution	70000	70000
MS/MS resolution	17500	17500
TopN	3	3
NCE/stepped NCE	20,40,60	20,40,60

### Collection and screening of active components

The compounds identified in LC-MS were further screened by SwissADME (http://www.swissadme.ch/) and ProTox-II (https://tox-new.charite.de/) to determine whether these identified compounds could be used for analysis. A chemical structure of each of these compounds was obtained from PubChem (https://pubchem.ncbi.nlm.nih.gov/), and the chemical structures were designed using InDraw (version 5.4.5).

### Network pharmacology

See the supplementary materials for the relevant analysis of network pharmacology and bioinformatics.

### Molecular docking

Molecular docking research was performed with the related software, AutoDockTools (Version 1.5.6), and the crystal structures of EGFR (PDB ID: 3POZ), AKT (PDB ID: 6CCY) and ALB (PDB ID: 4L8U) were downloaded from the RCSB Protein Date Bank. The ligand structures were downloaded from PubChem, and the docking procedure was repeated three times. Based on the docking results, the best scoring docked model was chosen for conformation, and Ligplot+ (Version 2.2.0) and PyMOL (Version 4.6.0) were used for visualization.

### Molecular dynamics (MD) simulation

MD simulations were conducted using GROMACS (version 2020.6). Interactions were modeled with the CHARMM36 force field using the TIP3P water model. The simulation system involved a cubic solvation box with the edge length set to 1.2 and the adjoint type periodic boundary condition to 1 ns. After solvation, ion equilibration was assigned to an ion concentration of 0.145 M to simulate the human environment and initial conformational equilibration. Then, the equilibration phase was completed in two steps: 100 ps in constant particle number, volume, and temperature ensemble. It was raised to 310 K and 1 bar, respectively, and finally, a 100 ns MD simulation was performed for the whole system. The nonbonded interaction cut-off value was set to 1.2 nm, and a PME algorithm was employed to determine the long-range electrostatic interactions. The time step was set to 2 fs, and the conformations were saved every 10 ps.

### Animals and experimental design

Adult male ICR mice weighing 18–20 g were acquired from Chengdu Dashuo Biotechnological Company (Chengdu, China). The standard feeding conditions of mice were as follows: 23 ± 2° C, humidity 55 ± 5%, and 12h light/dark cycle. The mice, taking standard laboratory feed and water at will, were randomly divided into 7 groups, including control, LPS/D-GalN, LPS/D-GalN + ESC (0.5 g/kg), LPS/D-GalN + ESC (1.5 g/kg), LPS/D-GalN + ESC (2.5 g/kg), LPS/D-GalN + silibinin (100 mg/kg), and ESC control (2.5 g/kg) groups, with 10 animals in each group. The mice were orally administered ESC, silibinin, or normal saline once a day for 7 days. On the 7th day, after administering ESC, silibinin, or normal saline for 1 h, the mice in LPS/D-GalN, LPS/D-GalN model + ESC (0.5, 1.5, or 2.5 g/kg), and LPS/D-GalN + silibinin groups were intraperitoneally injected with 600 mg/kg of D-GalN and 10 μg/kg of LPS; in contrast, the control and ESC control groups received injections of normal saline alone. The study flow has been detailed in Supplementary Figure 1. After 6 h, all the mice were sacrificed to obtain blood and tissue samples. The plasma was collected centrifuged at 3500 g for 10 min at 4° C to collect the supernatant, and placed at-80° C.

### Biochemistry analysis

The serum levels of alanine aminotransferase (ALT), aspartate aminotransferase (AST), total proteins (TP), and albumin (ALB) (all from Nanjing Jiancheng Bioengineering Institute, Nanjing, China) were tested based on the operation of the kit.

### Hematoxylin and eosin (H&E)

The liver tissue samples were excised from mice and fixed in 10% formaldehyde at 25° C. These tissues were embedded in paraffin, and 4 μm-thick transverse sections were cut, followed by HE staining. For each liver, a numerical score was assigned based on its degree of inflammation and necrosis. The scoring standard referred to previous research [37].

### TdT-mediated dUTP nick end labeling (TUNEL) staining analysis

TUNEL assay was carried out paraffin-embedded tissue with the TUNEL assay kit (Servicebio, Wuhan, China) based on the protocol provided.

### Detection of anti-inflammatory biomarkers

Serum levels of interleukin-6 (IL-6), interleukin-1β (IL-1β), and tumor necrosis factor-α (TNF-α) were measured using commercial enzyme-linked immunosorbent assay (ELISA) kits (all from FANKEWEI, Shanghai, China) following the manufacturer’s instructions.

### Analysis of the antioxidant system

Serum levels of Superoxide dismutase (SOD), malondialdehyde (MDA), glutathione (GSH), and catalase (CAT) were tested based on the operation of the kit (all from Solarbio Biological Technology Co., Ltd, Beijing, China).

### Cell cultures

HepG2 cells, acquired from Shanghai Fuheng Cell Center, were cultured in DMEM complete medium using 10% FBS, 1% penicillin, and streptomycin. The cells were placed in a 37° C, 5% CO2, and 95% air-humidified incubator.

### Cell viability assay

Cell viability was assessed with the CCK-8 assay (Solarbio China), based on the methods described by Ji et al. [38] Briefly, HepG2 cells were seeded in 96-well plates at a density of 5 × 104/mL and incubated for 24 h at 37° C and 5% CO2. Then, treated with LPS (1 μg/ml) and different doses of D-GalN or ESC for 24 h, 10 μl CCK-8 solution was put into every well and cultured under 37° C for 4 h. OD value of absorbance was measured at 450 nm.

### Cell death assessment

Calcein acetoxymethyl/propidium iodide (Calcein AM/PI) staining was utilized to evaluate live/dead cells. Calcein AM/PI staining was performed with a calcein AM/PI kit (from Shanghai Beyotime Biotechnology Co., Ltd, Beijing, China). The stained cells were viewed using a fluorescence microscope (Olympus, Japan).

### Cell apoptosis analysis by flow cytometry

HepG2 cells were seeded into 6-well plates at a density of 5 × 104/mL and incubated for 24 h. Then pretreated with ESC at different concentrations (0, 6.25, 12.5, 25 μg/mL) for 24 h, next, the cells were treated with LPS/D-GalN for 24 h and harvested. Afterward, the cells were resuspended in 500 μl binding buffer and stained with 5ul Annexin V (AV)-FITC, 5ul PI using an AV-FITC/PI staining kit (from KeyGene BioTech, China). The status of cell apoptosis was analyzed by flow cytometry (Bio-Rad, USA).

### ALT, AST, and inflammatory factors measurement

Based on the manufacturers’ instructions, the cell culture supernatant cytokine (IL-1β, IL-6, TNF-α) concentrations were determined with a commercially available ELISA kit (Shanghai Hengyuan Biological Technology CO., Ltd, Shanghai China). ALT and AST level in cell culture supernatant were determined using ALT and AST assay kits.

### Intracellular ROS assay

The generation of intracellular ROS of HepG2 cells was determined with a DCFH-DA kit (Solarbio, China). After LPS/D-GalN treatment for 24 h, the cells were stained using 10 μM DCFH-DA and incubated for 20 min. The pictures were obtained using fluorescence microscopy.

### Mitochondria membrane potential (MMP) assay

The MMP was determined using a JC-10 assay kit (Solarbio, China). First, the cells were treated as previously described within the text. Then, JC-10 staining was performed based on the manufacturer’s instructions.

### Real-time PCR assay

RNA was isolated from cells using TRIzol Reagent (Sangon Biotech Co., Ltd., Shanghai, China), and cDNA was synthesized with a cDNA Kit. The expression of the indicated genes was analyzed using qRT-PCR amplified with SYBR Green (all from Transgene, China). The relative expression levels were determined using the 2^-ΔΔCq^ method and normalized to the internal reference gene GAPDH. The primer sequences are observed in Table 2.

**Table 2:** Primers’ list.

**Gene**	**Primer sequence**	
	**Forward (5’-3’)**	**Reverse (5 -3’)**
AKT1	AGCGACGTGGCTATTGTGAAG	GCCATCATTCTTGAGGAGGAAGT
EGFR	AGGCACGAGTAACAAGCTCAC	ATGAGGACATAACCAGCCACC
VEGFA	AGGCACGAGTAACAAGCTCAC	ATGAGGACATAACCAGCCACC
ALB	GCAGATGACAGGGCGGAACTTG	ACAGTGGGCTTTCTTCAACAGTGG
MTOR	GCAGATTTGCCAACTATCTTCGG	CAGCGGTAAAAGTGTCCCCTG
HIF1A	TTCCCGACTAGGCCCATTC	CAGGTATTCAAGGTCCCATTTCA
ERBB2	TGCAGGGAAACCTGGAACTC	ACAGGGGTGGTATTGTTCAGC
GAPDH	ACAACTTTGGTATCGTGGAAGG	GCCATCACGCCACAGTTTC

### Western blot

The cells were treated and collected using trypsin-EDTA. The HepG2 cells were homogenized in a modified RIPA buffer (Solarbio, Beijing, China) using phenyl methane sulfonyl fluoride (Amresco, USA) and a 1 × cocktail protease inhibitor (Beyotime, Shanghai, China) to extract protein. The supernatants were collected after centrifugation at 12,000 rpm for 15 min at 4° C. Protein concentrations were quantified using a BCA protein assay kit. Nuclear and cytoplasmic protein extraction was performed with a nuclear and cytoplasmic protein extraction kit (all from Beyotime, Shanghai, China). SDS-PAGE gels were utilized to separate the proteins, which were then transferred to polyvinylidene difluoride membranes. Afterward, the membranes were blocked with 5% non-fat dry milk in TBST for 2 h at room temperature. The primary antibody was diluted using TBST and added to protein samples for overnight incubation in a refrigerator at 4° C. After being washed three times using TBST, the membranes were incubated using their secondary antibodies conjugated with horseradish peroxidase. Finally, the membranes were examined by exposing them to an ECL reagent.

### Statistical analysis

Statistical tests (t-test and ANOVA) were performed using GraphPad Prism (version 8.0). The data are expressed as mean ± SD. Fluorescence intensity and gray scale values were analyzed using image J (version 1.53n). A p-value of < 0.05 was determined to be statistically significant.

### Data availability

All the data can be obtained from the corresponding author upon request.

## Supplementary Material

Supplementary Methods

Supplementary Figure 1

Supplementary Tables 1-5

Supplementary Table 6
