# Supplementary material for: Swertia cincta Burkill alleviates LPS/D-GalN-induced acute liver failure by modulating apoptosis and oxidative stress signaling pathways
Source: Aging (Albany NY). 2023 Jun 27;15(12):5887–916. doi: 10.18632/aging.204848 (PMC10333062; doi:10.18632/aging.204848)
Supplement: Supplementary Methods [file aging-15-204848-s001.pdf]

SUPPLEMENTARY MATERIALS

Supplementary Methods

Prediction of ESC targets

The simplified molecular-input line-entry system (SMILE) of candidate compounds, previously described in ESC, selected in the previous step, was retrieved from PubChem, DrugBank (https://go.drugbank.com/), or ChEMBL (https://www.ebi.ac.uk/chembl/) servers for further analysis. After that, we uploaded canonical SMILES to the SwissTargetPrediction database (http://www.swisstargetprediction.ch/) and Sea database (https://sea.bkslab.org/) to predict ESC targets. Simultaneously, the HERB database (http://herb.ac.cn/) was utilized to search and predict the targets of these candidate compounds. Finally, the probe ID was converted to the gene SYMBOL name in the downloaded file using the R software.

Predict targets of ESC against ALF

Genecard (https://www.genecards.org/), OMIM (https://www.omim.org/), TTD (http://db.idrblab.net/ttd/), PharmGKB (https://www.pharmgkb.org/), and DrugBank (https://go.drugbank.com/) databases were searched using the keywords "acute liver failure" and "acute liver injury." Then, the predicted targets were standardized using the UniProt database (http://www.uniprot.org/uniprot/) and corrected to their official gene names.

Screening of intersection targets

We intersected the potential targets of ALF and ESC-related drug targets to illustrate the potential interaction of ESC-related targets with ALF targets. We obtained the intersecting targets using jvenn (http://jvenn.toulouse.inra.fr/), and a protein class analysis was performed depending on the PANTHER classification system (http://www.pantherdb.org/).

Network construction and analysis

Among these potential targets, the protein-protein interaction (PPI) was constructed with the STRING database (https://string-db.org/). The species was limited to "Homo sapiens" with a confidence score >0.4. Consequently, the STRING network was exported to txt format and imported within Cytoscape (Version 3.8.0) for network visualization using available tutorials for implementing Cytoscape (https://cytoscape.org/). NetworkAnalyzer (http://apps.cytoscape.org/apps/networkanalyzer) was used to analyze degree distribution, clustering coefficient, and edge betweenness centrality using Cytoscape plugins. A higher degree value node depicted putative crucial targets of ESC against ALF in the PPI network. Finally, the top 10 targets of intersection targets were selected for the subsequent study.

Analysis by gene ontology (GO) and the Kyoto encyclopedia of genes and genomes (KEGG) pathway

GO and KEGG pathway enrichment analyses were performed using R software (Version 4.1.1) through the "clusterprofiler" package. GO terms and relevant pathways were selected as significant signaling pathways with a P value<0.05. Using an online tool, we visualized the top 20 significant terms for GO and KEGG analysis (http://www.bioinformatics.com.cn/).

Verification of key targets in gene expression omnibus (GEO) database

The expression of the key genes screened from PPI was verified using the GEO database (https://www.ncbi.nlm.nih.gov/geo/). The keywords were set as "acute liver failure," "acute liver injury," and "homo sapiens." The tissue type was limited to liver tissue. Depending on the filter criteria, the expression data of GSE38941 were downloaded. GPL570 was the platform of the dataset, which included 17 ALF samples and 10 controls. The probe ID was converted to a gene symbol with the R software for further analysis.
