# Supplementary figures and images for: Swertia cincta Burkill alleviates LPS/D-GalN-induced acute liver failure by modulating apoptosis and oxidative stress signaling pathways
Source: Aging (Albany NY). 2023 Jun 27;15(12):5887–916. doi: 10.18632/aging.204848 (PMC10333062; doi:10.18632/aging.204848)

SUPPLEMENTARY FIGURE

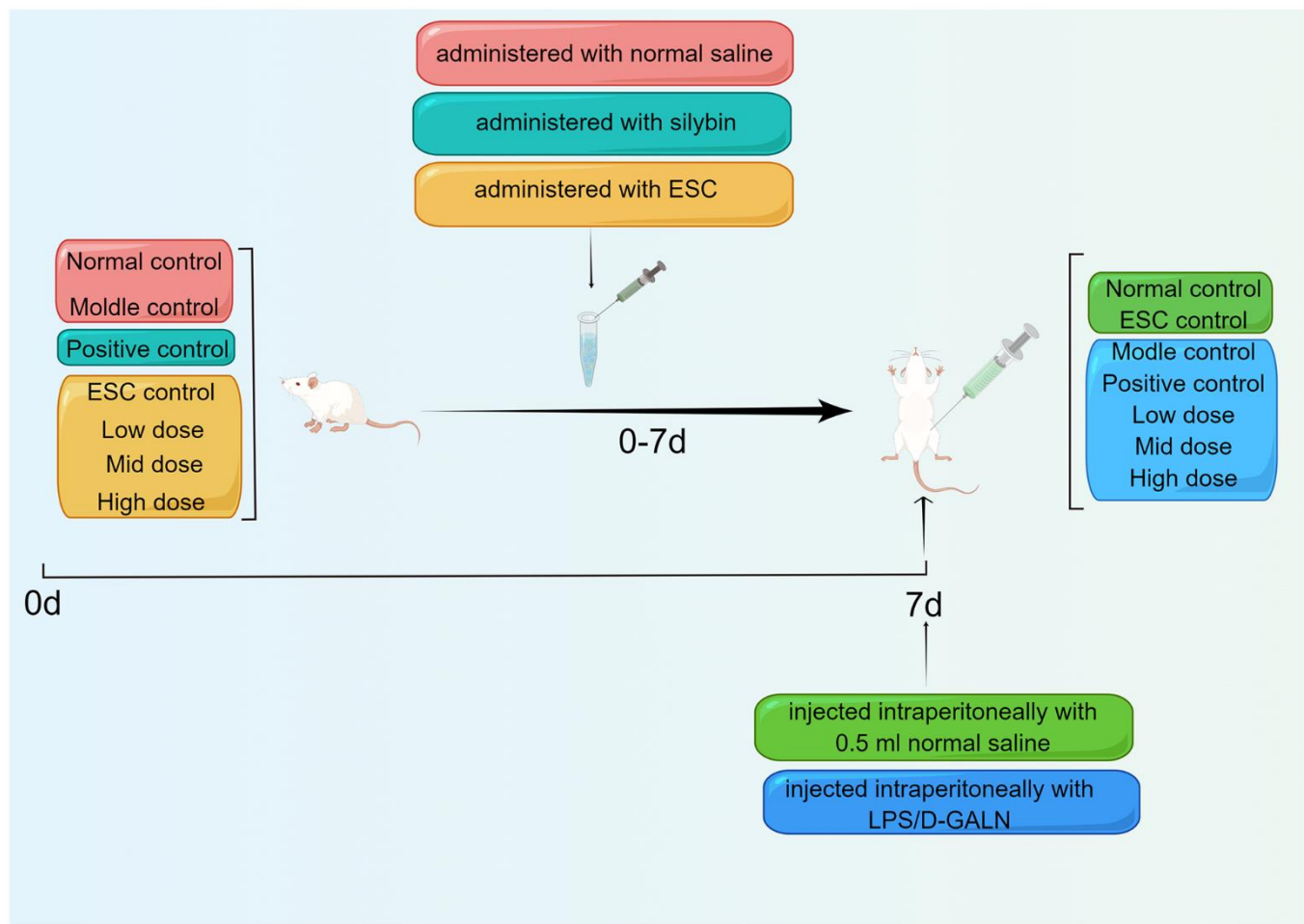

Supplementary Figure 1. Animal experimental procedure diagram.

Supplement: Supplementary Figure 1 [file aging-15-204848-s002.pdf]
