# Supplementary material for: Swertia cincta Burkill alleviates LPS/D-GalN-induced acute liver failure by modulating apoptosis and oxidative stress signaling pathways
Source: Aging (Albany NY). 2023 Jun 27;15(12):5887–916. doi: 10.18632/aging.204848 (PMC10333062; doi:10.18632/aging.204848)
Supplement: Supplementary Tables 1-5 [file aging-15-204848-s003.pdf]

SUPPLEMENTARY TABLES

Supplementary Table 1. The ESC freeze-dry powder qualitative analysis results.

No.	RT(min)	Identification	Observed(*m/z*)	Adduct	Diff.(ppm)	Formula	Chemical class
1	2.67	Geniposidic acid	373.1127	[M-H]	3.45	C16H22O10	Iridoids
2	2.78	Gentisic acid	153.0194	[M-H]	2.87	C7H6O4	Xanthones
3	2.83	loganic acid	375.1301	[M-H]-	2.97	C16H24O10	Organic acids and derivatives
4	3.49	Asperulosidic acid	431.1186	[M-H]	0.94	C18H24O12	Iridoids
5	3.51	Swertiamarin	419.1189	[M+HCOO]	0.17	C16H22O10	Iridoids
6	4.09	Gentianine	176.0704	[M+H]+	2.08	C10H9NO2	Alkaloids
7	4.25	Isomangiferin	421.0775	[M-H]	1.14	C19H18O11	Xanthones
8	4.41	Sweroside	403.1243	[M+HCOO]	0.80	C16H22O9	Terpenoids
9	4.96	4-Methylumbelliferone	175.0399	[M-H]	0.75	C10H8O3	Coumarins and derivatives
10	4.98	Homoorientin	447.0921	[M-H]	1.96	C21H20O11	Flavonoids
11	5.05	Gentiopicroside	357.1170	[M+H]+	0.03	C16H20O9	Iridoids
12	5.57	Rutin	611.1586	[M+H]+	2.34	C27H30O16	Flavonoids
13	5.62	Isovitexin	431.0990	[M-H]	2.38	C21H20O10	Flavonoids
14	5.63	Apigenin-8-C-glucoside	433.1127	[M+H]+	0.68	C21H20O10	Flavonoids
15	5.66	Hyperoside	465.1029	[M+H]	2.02	C21H20O12	Flavonoids
16	5.89	Verbenalin	433.1342	[M+HCOO]	1.96	C17H24O10	Iridoids
17	6.06	kaempferol 7-neohesperidoside	595.1669	[M+H]+	1.59	C27H30O15	Flavonoids
18	6.56	4-Methoxysalicylic acid	167.0349	[M-H]	0.40	C8H8O4	Phenols
19	6.65	Luteolin-4'-O-glucoside	447.0932	[M-H]-	0.51	C21H20O11	Flavonoids
20	6.66	Isoorientin	449.1073	[M+H]+	1.61	C21H20O11	Flavonoids
21	6.72	3-hydroxy-4-methoxyxanthen-9-one	243.0650	[M+H]+	0.20	C14H10O4	Miscellaneous
22	6.78	(2R,3R)-3,5-dihydroxy-2-(4-hydroxyphenyl)-7-methoxy-2,3-dihydrochromen-4-one	303.0865	[M+H]+	1.57	C16H14O6	Flavonoids
23	6.92	Iridin	521.1293	[M-H]	1.37	C24H26O13	Flavonoids
24	7.09	Amarogentin	587.1769	[M+H]	1.49	C29H30O13	Iridoids
25	7.15	Dihydrokaempferol	289.0702	[M+H]+	0.81	C15H12O6	Flavonoids
26	7.79	Syringaresinol	419.1708	[M+H]+	1.99	C22H26O8	Phenylpropanoids
27	8.41	(3-hydroxyphenyl)-(2,4,6-trihydroxyphenyl)methanone	247.0598	[M+H]+	0.82	C13H10O5	Miscellaneous
28	8.45	Luteolin	287.0554	[M+H]	1.38	C15H10O6	Flavonoids
	8.53	Kaempferide	301.0708	[M+H]+	0.56	C16H12O6	Flavonoids
29	8.97	1,5,8-trihydroxy-3-methoxyxanthen-9-one	275.0546	[M+H]+	1.38	C14H10O6	Miscellaneous
30	9.84	Tetrahydroxyxanthone	259.0252	[M-H]	0.94	C13H8O6	Flavonoids
31	9.84	(2S,3S)-2-(3,4-dihydroxyphenyl)-3,7-dihydroxy-2,3-dihydrochromen-4-one	289.0704	[M+H]+	1.46	C15H12O6	Flavonoids
32	10.24	trifolirhizin	469.1120	[M+Na]+	0.02	C22H22O10	Isoflavonoids
33	10.44	Oleanonic acid	455.3511	[M+H]	2.03	C30H46O3	Terpenoids
34	10.51	Eriodictyol	289.0704	[M+H]	1.25	C15H12O6	Flavonoids
35	11.27	Maslinic acid	473.3626	[M+H]+	0.82	C30H48O4	Terpenoids
36	12.06	Kaempferol	285.0402	[M-H]-	0.64	C15H10O6	Flavonoids
37	14.87	Oleanolic acid	455.3527	[M-H]-	0.74	C30H48O3	Terpenoids
38	16.05	Genistein	269.0455	[M-H]-	2.01	C15H10O5	Flavonoids
39	16.81	3-Epilupeol	427.3941	[M+H]+	0.13	C30H50O	Terpenoids
40	18.00	Betulinic acid	455.3527	[M-H]-	0.61	C30H48O3	Terpenoids
41	21.63	Ursolic acid	439.3562	[M+H-H2O]-	1.81	C30H48O3	Terpenoids

Supplementary Table 2. The targets in ESC for ALF treatment.

Number	Gene ID	Protein description	Gene symbol
1	2147	coagulation factor II	F2
2	213	albumin	ALB
3	4233	MET proto-oncogene, receptor tyrosine kinase	MET
4	5788	protein tyrosine phosphatase receptor type C	PTPRC
5	6554	solute carrier family 10 member 1	SLC10A1
6	975	CD81 molecule	CD81
7	3717	Janus kinase 2	JAK2
8	7297	tyrosine kinase 2	TYK2
9	5468	peroxisome proliferator activated receptor gamma	PPARG
10	3558	interleukin 2	IL2
11	3643	insulin receptor	INSR
12	1956	epidermal growth factor receptor	EGFR
13	7422	vascular endothelial growth factor A	VEGFA
14	6772	signal transducer and activator of transcription 1	STAT1
15	2322	fms related receptor tyrosine kinase 3	FLT3
16	7015	telomerase reverse transcriptase	TERT
17	3815	KIT proto-oncogene, receptor tyrosine kinase	KIT
18	5290	phosphatidylinositol-4,5-bisphosphate 3-kinase catalytic subunit alpha	PIK3CA
19	25	ABL proto-oncogene 1, non-receptor tyrosine kinase	ABL1
20	1557	cytochrome P450 family 2 subfamily C member 19	CYP2C19
21	4353	myeloperoxidase	MPO
22	5054	serpin family E member 1	SERPINE1
23	5243	ATP binding cassette subfamily B member 1	ABCB1
24	20186	nuclear receptor subfamily 1, group H, member 4	NR1H4
25	4318	matrix metallopeptidase 9	MMP9
26	18126	nitric oxide synthase 2, inducible	NOS2
27	1991	elastase, neutrophil expressed	ELANE
28	217	aldehyde dehydrogenase 2 family member	ALDH2
29	5781	protein tyrosine phosphatase non-receptor type 11	PTPN11
30	2168	fatty acid binding protein 1	FABP1
31	596	BCL2 apoptosis regulator	BCL2
32	1080	CF transmembrane conductance regulator	CFTR
33	383	arginase 1	ARG1
34	7276	transthyretin	TTR
35	5836	glycogen phosphorylase L	PYGL
36	5747	prostaglandin-endoperoxide synthase 2	PTGS2
37	3716	Janus kinase 1	JAK1
38	207	AKT serine/threonine kinase 1	AKT1
39	4193	MDM2 proto-oncogene	MDM2
40	2321	fms related receptor tyrosine kinase 1	FLT1
41	3091	hypoxia inducible factor 1 subunit alpha	HIF1A
42	3465	peroxisome proliferator activated receptor alpha	PPARA
43	2539	glucose-6-phosphate dehydrogenase	G6PD
44	23411	sirtuin 1	SIRT1
45	2475	mechanistic target of rapamycin kinase	MTOR
46	5327	plasminogen activator, tissue type	PLAT
47	5159	platelet derived growth factor receptor beta	PDGFRB
48	2629	glucosylceramidase beta 1	GBA

49	5340	plasminogen	PLG
50	4282	macrophage migration inhibitory factor	MIF
51	6403	selectin P	SELP
52	4311	membrane metalloendopeptidase	MME
53	2260	fibroblast growth factor receptor 1	FGFR1
54	22259	nuclear receptor subfamily 1, group H, member 3	NR1H3
55	598	BCL2 like 1	BCL2L1
56	4363	ATP binding cassette subfamily C member 1	ABCC1
57	2099	estrogen receptor 1	ESR1
58	3251	hypoxanthine phosphoribosyltransferase 1	HPRT1
59	7498	xanthine dehydrogenase	XDH
60	100	adenosine deaminase	ADA
61	3791	kinase insert domain receptor	KDR
62	6850	spleen associated tyrosine kinase	SYK
63	673	B-Raf proto-oncogene, serine/threonine kinase	BRAF
64	4153	myoglobin	MB
65	116085	solute carrier family 22 member 12	SLC22A12
66	9429	ATP binding cassette subfamily G member 2	ABCG2
67	6555	solute carrier family 10 member 2	SLC10A2
68	5319	phospholipase A2 group IB	PLA2G1B
69	2064	erb-b2 receptor tyrosine kinase 2	ERBB2
70	5604	mitogen-activated protein kinase kinase 1	MAP2K1
71	351	amyloid beta precursor protein	APP
72	6469	sonic hedgehog signaling molecule	SHH

Supplementary Table 3. The top 10 GO terms of BP, MF, and CC.

ONTOLOGY	ID	Description	geneID	Count
BP	GO:0018108	peptidyl-tyrosine phosphorylation	MET/PTPRC/CD81/JAK2/TYK2/IL2/INSR/EGFR/VEGFA/FLT3/KIT/ABL1/PTPN11/JAK1/FLT1/MTOR/PDGFRB/MIF/FGFR1/KDR/SYK/ERBB2/MAP2K1/APP	24
BP	GO:0018212	peptidyl-tyrosine modification	MET/PTPRC/CD81/JAK2/TYK2/IL2/INSR/EGFR/VEGFA/FLT3/KIT/ABL1/PTPN11/JAK1/FLT1/MTOR/PDGFRB/MIF/FGFR1/KDR/SYK/ERBB2/	
MAP2K1/APP	24			
BP	GO:0014065	phosphatidylinositol 3-kinase signaling	F2/JAK2/INSR/EGFR/FLT3/KIT/PIK3CA/AKT1/FLT1/SIRT1/PDGFRB/SELP/FGFR1/KDR/ERBB2	15
BP	GO:0071902	positive regulation of protein serine/threonine kinase activity	PTPRC/CD81/INSR/EGFR/VEGFA/FLT3/KIT/ELANE/PTPN11/AKT1/FLT1/SIRT1/PDGFRB/FGFR1/SYK/PLA2G1B/ERBB2/MAP2K1	18
BP	GO:0043406	positive regulation of MAP kinase activity	PTPRC/CD81/INSR/EGFR/VEGFA/FLT3/KIT/ELANE/PTPN11/FLT1/PDGFRB/FGFR1/SYK/PLA2G1B/ERBB2/MAP2K1	16
BP	GO:0045834	positive regulation of lipid metabolic process	F2/CD81/PPARG/FLT3/KIT/NR1H4/FABP1/PTGS2/AKT1/FLT1/PPARA/MTOR/PDGFRB/NR1H3	14
BP	GO:0048015	phosphatidylinositol-mediated signaling	F2/JAK2/INSR/EGFR/FLT3/KIT/PIK3CA/AKT1/FLT1/SIRT1/PDGFRB/SELP/FGFR1/KDR/ERBB2	15
BP	GO:0048017	inositol lipid-mediated signaling	F2/JAK2/INSR/EGFR/FLT3/KIT/PIK3CA/AKT1/FLT1/SIRT1/PDGFRB/SELP/FGFR1/KDR/ERBB2	15
BP	GO:0014068	positive regulation of phosphatidylinositol 3-kinase signaling	F2/JAK2/INSR/FLT3/KIT/PIK3CA/FLT1/SIRT1/PDGFRB/SELP/FGFR1/KDR	12
BP	GO:0071900	regulation of protein serine/threonine kinase activity	PTPRC/CD81/INSR/EGFR/VEGFA/FLT3/KIT/ABL1/ELANE/PTPN11/AKT1/FLT1/SIRT1/PDGFRB/GBA/FGFR1/SYK/PLA2G1B/ERBB2/MAP2K1	20
CC	GO:0031983	vesicle lumen	ALB/EGFR/VEGFA/MPO/SERPINE1/ELANE/ARG1/TTR/PYGL/PLG/MIF/ADA/APP	13
CC	GO:0060205	cytoplasmic vesicle lumen	ALB/VEGFA/MPO/SERPINE1/ELANE/ARG1/TTR/PYGL/PLG/MIF/ADA/APP	12
CC	GO:0034774	secretory granule lumen	ALB/VEGFA/MPO/SERPINE1/ELANE/ARG1/TTR/PYGL/PLG/MIF/APP	11
CC	GO:0045177	apical part of cell	EGFR/ABCB1/FABP1/CFTR/PDGFRB/ABCC1/SLC22A12/ABCG2/SLC10A2/ERBB2/APP	11
CC	GO:0031091	platelet alpha granule	ALB/VEGFA/SERPINE1/PLG/SELP/APP	6
CC	GO:0045121	membrane raft	PTPRC/JAK2/INSR/EGFR/MME/KDR/ABCG2/APP/SHH	9
CC	GO:0098857	membrane microdomain	PTPRC/JAK2/INSR/EGFR/MME/KDR/ABCG2/APP/SHH	9
CC	GO:0031093	platelet alpha granule lumen	ALB/VEGFA/SERPINE1/PLG/APP	5
CC	GO:0016324	apical plasma membrane	EGFR/ABCB1/CFTR/PDGFRB/ABCC1/SLC22A12/ABCG2/SLC10A2/ERBB2	9
CC	GO:0009897	external side of plasma membrane	F2/PTPRC/INSR/KIT/ABCB1/PLG/SELP/ADA/ABCG2	9
MF	GO:0004713	protein tyrosine kinase activity	MET/JAK2/TYK2/INSR/EGFR/FLT3/KIT/ABL1/JAK1/FLT1/PDGFRB/FGFR1/KDR/SYK/ERBB2/MAP2K1	16
MF	GO:0004714	transmembrane receptor protein tyrosine kinase activity	MET/INSR/EGFR/FLT3/KIT/FLT1/PDGFRB/FGFR1/KDR/ERBB2	10
MF	GO:0019199	transmembrane receptor protein kinase activity	MET/INSR/EGFR/FLT3/KIT/FLT1/PDGFRB/FGFR1/KDR/ERBB2	10
MF	GO:0051427	hormone receptor binding	JAK2/TYK2/PPARG/STAT1/FLT3/NR1H4/PTPN11/JAK1/HIF1A/SIRT1/ESR1	11
MF	GO:0043560	insulin receptor substrate binding	JAK2/INSR/PIK3CA/PTPN11	4
MF	GO:0019902	phosphatase binding	MET/PPARG/EGFR/BCL2/JAK1/AKT1/PPARA/SYK/ERBB2	9
MF	GO:0019838	growth factor binding	INSR/EGFR/FLT3/FLT1/PDGFRB/FGFR1/KDR/ERBB2	8
MF	GO:0032052	bile acid binding	NR1H4/FABP1/PYGL/PLA2G1B	4
MF	GO:0008201	heparin binding	F2/PTPRC/VEGFA/MPO/ELANE/SELP/FGFR1/APP	8
MF	GO:0005539	glycosaminoglycan binding	F2/PTPRC/VEGFA/MPO/ELANE/SELP/FGFR1/APP/SHH	9

Supplementary Table 4. The top 20 enriched KEGG pathways.

ID	Description	GeneRatio	geneID	Count
hsa01521	EGFR tyrosine kinase inhibitor resistance	15/70	MET/JAK2/EGFR/VEGFA/PIK3CA/BCL2/JAK1/AKT1/MTOR/PDGFRB/BCL2L1/KDR/BRAF/ERBB2/MAP2K1	15
hsa05230	Central carbon metabolism in cancer	13/70	MET/EGFR/FLT3/KIT/PIK3CA/AKT1/HIF1A/G6PD/MTOR/PDGFRB/FGFR1/ERBB2/MAP2K1	13
hsa04151	PI3K-Akt signaling pathway	22/70	MET/JAK2/IL2/INSR/EGFR/VEGFA/FLT3/KIT/PIK3CA/BCL2/JAK1/AKT1/MDM2/FLT1/MTOR/PDGFRB/FGFR1/BCL2L1/KDR/SYK/ERBB2/MAP2K1	22
hsa05205	Proteoglycans in cancer	17/70	MET/EGFR/VEGFA/PIK3CA/MMP9/PTPN11/AKT1/MDM2/HIF1A/MTOR/FGFR1/ESR1/KDR/BRAF/ERBB2/MAP2K1/SHH	17
hsa05215	Prostate cancer	13/70	EGFR/PIK3CA/MMP9/BCL2/AKT1/MDM2/MTOR/PLAT/PDGFRB/FGFR1/BRAF/ERBB2/MAP2K1	13
hsa04066	HIF-1 signaling pathway	13/70	INSR/EGFR/VEGFA/PIK3CA/SERPINE1/NOS2/BCL2/AKT1/FLT1/HIF1A/MTOR/ERBB2/MAP2K1	13
hsa04014	Ras signaling pathway	17/70	MET/INSR/EGFR/VEGFA/FLT3/KIT/PIK3CA/ABL1/PTPN11/AKT1/FLT1/PDGFRB/FGFR1/BCL2L1/KDR/PLA2G1B/MAP2K1	17
hsa05212	Pancreatic cancer	11/70	EGFR/VEGFA/STAT1/PIK3CA/JAK1/AKT1/MTOR/BCL2L1/BRAF/ERBB2/MAP2K1	11
hsa01522	Endocrine resistance	11/70	EGFR/PIK3CA/MMP9/BCL2/AKT1/MDM2/MTOR/ESR1/BRAF/ERBB2/MAP2K1	11
hsa04630	JAK-STAT signaling pathway	13/70	JAK2/TYK2/IL2/EGFR/STAT1/PIK3CA/PTPN11/BCL2/JAK1/AKT1/MTOR/PDGFRB/BCL2L1	13
hsa05235	PD-L1 expression and PD-1 checkpoint pathway in cancer	10/70	JAK2/EGFR/STAT1/PIK3CA/PTPN11/JAK1/AKT1/HIF1A/MTOR/MAP2K1	10
hsa05226	Gastric cancer	12/70	MET/EGFR/TERT/PIK3CA/ABCB1/BCL2/AKT1/MTOR/BRAF/ERBB2/MAP2K1/SHH	12
hsa05206	MicroRNAs in cancer	16/70	MET/EGFR/VEGFA/PIK3CA/ABL1/ABCB1/MMP9/BCL2/PTGS2/MDM2/SIRT1/MTOR/PDGFRB/ABCC1/ERBB2/MAP2K1	16
hsa05218	Melanoma	9/70	MET/EGFR/PIK3CA/AKT1/MDM2/PDGFRB/FGFR1/BRAF/MAP2K1	9
hsa04015	Rap1 signaling pathway	13/70	MET/INSR/EGFR/VEGFA/KIT/PIK3CA/AKT1/FLT1/PDGFRB/FGFR1/KDR/BRAF/MAP2K1	13
hsa04072	Phospholipase D signaling pathway	11/70	F2/INSR/EGFR/KIT/PIK3CA/PTPN11/AKT1/MTOR/PDGFRB/SYK/MAP2K1	11
hsa05219	Bladder cancer	7/70	EGFR/VEGFA/MMP9/MDM2/BRAF/ERBB2/MAP2K1	7
hsa05167	Kaposi sarcoma-associated herpesvirus infection	12/70	JAK2/TYK2/VEGFA/STAT1/PIK3CA/PTGS2/JAK1/AKT1/HIF1A/MTOR/SYK/MAP2K1	12
hsa05160	Hepatitis C	11/70	CD81/TYK2/EGFR/STAT1/PIK3CA/JAK1/AKT1/PARA/NR1H3/BRAF/MAP2K1	11

Supplementary Table 5. Volatile components in ESC.

No.	Name	MW	Results
1	3-methyl-cyclopentanol	100.16	Accepted
2	4-hydroxy-4-methyl-2-pentanone	116.16	Accepted
3	(Z) -3-hexen-1-ol	299.99	Accepted
4	ethyl benzene	106.17	Accepted
5	2,4-Hexadienal	96.13	Accepted
6	1-methylethyl-benzene	120.19	Accepted
7	1-octen-3-ol	128.21	Accepted
8	pentanoic acid	102.13	Accepted
9	octyl aldehyde	128.21	Accepted
10	(2E,4E,6E)-octa-2,4,6-trienal	122.16	Accepted
11	Phenylacetaldehyde	120.15	Accepted
12	(-)-Linalool	145.25	Accepted
13	nonyl aldehyde	142.24	Accepted
14	L-α-terpineol	154.25	Accepted
15	decanal	156.26	Accepted
16	Bicyclo[5.2.0]non-1-ene	122.21	Accepted
17	3-Ethyl-4-methyl-1H-pyrrole-2,5-dione	139.15	Accepted
18	Cyclohexane	124.22	Accepted
19	geraniol	154.25	Accepted
20	swertiol	154.21	Accepted
21	1-nonanol	144.25	Accepted
22	3,3,6-trimethyl-1,5-heptadien-4-one	152.23	Accepted
23	Bicycle[3.2.1]octan-3-one	124.18	Accepted
24	(Z) -2-pentadecen-4-yne	206.37	Accepted
25	(Z) -9-hydroxy-4-methyl-7-nonenoic acid lactone	168.23	Accepted
26	1-undecyne	152.28	Accepted
27	4-vinyl-2-methoxy-phenol	150.17	Accepted
28	(E, E) -2,4-decadienal	152.23	Accepted
	2,3-dihydro-4H-1-benzopyran-4-one	148.16	Accepted
29	1,2-dimethyl-1,5-cyclooctadiene	136.23	Accepted
30	2-methoxy-4-(2-propenyl) -phenol	164.20	Accepted
31	nerolic acid	162.18	Accepted
32	1-dodecanol	186.33	Accepted
33	cyanic acid-2,4-dimethylphenyl ester	147.17	Accepted
34	(E) -6,10-dimethyl-5,9-undecadien-2-one	194.31	Accepted
35	(E) -2-methoxy-4-(1-propenyl) -phenol	164.20	Accepted
36	2-methyl-decane	156.31	Accepted
37	1,3,5,7-tetramethyl-adamantane	192.34	Accepted
38	2-methyl-5-(1,1,5-trimethyl-5-hexenyl) -furan	206.32	Accepted
39	pentadecane	212.41	Accepted
40	3-decen-5-one	154.25	Accepted
41	cis-hexahydro-8a-methyl-1,8(2H,5H) -naphthalenedione	180.24	Accepted
42	benzoic acid-4-ethoxy ethyl ester	194.23	Accepted
43	5,6,7,7a-tetrahydro-4,4,7a-trimethyl-2(4H) -benzofuranone	180.24	Accepted
44	octadecane	254.49	Accepted
45	cedryl propyl ether	264.44	Accepted
46	2-(acetyloxy) -1,4-benzene-dicarboxylic acid-dimethyl ester	252.22	Accepted
47	3,4-dihydro-1-oxo-1H-2-benzopyran-5-carboxaldehyde	176.17	Accepted

48	tetradecanal	212.37	Accepted
49	heneicosane	296.57	Accepted
50	(Z) -10-methyl-11-tetradecen-1-ol propionate	282.46	Accepted
51	isopropyl myristate	270.45	Accepted
52	5,9,13-trimethyl-4,8,12-tetradecatrienal	248.40	Accepted
53	6,10,14-trimethyl-2-pentadecanone	268.48	Accepted
54	tetrakis(1-methylethylidene) -cyclobutane	192.34	Accepted
55	dibutyl phthalate	278.34	Accepted
56	nonadecane	268.52	Accepted
57	1,2-benzene-dicarboxylic acid-butyl-8-methylnonyl ester	362.50	Accepted
58	[(E,E) -3,7,11-trimethyl-2,6,10-dodecatrien-1-yl]esterbenzoic acid	326.47	Accepted
59	methyl 13-methyltetradecanoate	256.42	Accepted
60	ester with butyl Glycolate-butylester-phthalic acid	336.38	Accepted
61	hexadecanoic acid-ethyl ester	284.48	Accepted
62	eicosane	282.55	Accepted
63	docosane	310.60	Accepted
64	pentacosane	352.68	Accepted
65	phytol isomer	296.53	Accepted
66	(E,E,Z) -1,3,12-nonadecatriene-5,14-diol	294.47	Accepted
67	trans-2-nonadecene	266.50	Accepted
68	hexacosane	366.70	Accepted
69	tricosane	324.63	Accepted
70	1-dotriacontanol	466.86	Accepted
71	(Z) -14-tricosenyl formate	366.62	Accepted
72	pentatriacontane	492.94	Accepted
73	phthalic acid-diisooctyl ester	390.55	Accepted
