# Supplementary material for: Swertia cincta Burkill alleviates LPS/D-GalN-induced acute liver failure by modulating apoptosis and oxidative stress signaling pathways
Source: Aging (Albany NY). 2023 Jun 27;15(12):5887–916. doi: 10.18632/aging.204848 (PMC10333062; doi:10.18632/aging.204848)
Supplement: Supplementary Table 6 [file aging-15-204848-s004.docx]

**Supplementary Table 6.** Evaluation of ADME properties of compounds in ESC.

| No. | Name | Structure | MW | Hdon | Hacc | AlogP | TPSA | Bioavailability Score | Lipinski rules  violation | Results |
| --- | --- | --- | --- | --- | --- | --- | --- | --- | --- | --- |
| 1 | Geniposidic acid |  | 374.38 | 6 | 10 | -2.50 | 166.14 | 0.11 | 1 | Accepted |
| 2 | Gentisic acid |  | 154.13 | 3 | 4 | 0.90 | 77.76 | 0.56 | 0 | Accepted |
| 3 | Loganic acid |  | 376.40 | 6 | 10 | -2.33 | 166.14 | 0.11 | 1 | Accepted |
| 4 | Asperulosidic acid |  | 432.42 | 6 | 12 | -3.16 | 192.44 | 0.11 | 2 | Rejected |
| 5 | Swertiamarin |  | 374.38 | 5 | 10 | -2.57 | 155.14 | 0.11 | 0 | Accepted |
| 6 | Gentianine |  | 175.20 | 0 | 3 | 1.16 | 39.19 | 0.55 | 0 | Accepted |
| 7 | Isomangiferin |  | 422.37 | 8 | 11 | -0.50 | 201.28 | 0.17 | 2 | Rejected |
| 8 | Sweroside |  | 358.34 | 4 | 9 | -1.33 | 134.91 | 0.56 | 0 | Accepted |
| 9 | 4-Methylumbelliferone |  | 176.18 | 1 | 3 | 2.08 | 0.55 | 0.55 | 0 | Accepted |
| 10 | Homoorientin |  | 448.41 | 8 | 11 | -0.32 | 201.28 | 0.17 | 2 | Rejected |
| 11 | Gentiopicroside |  | 356.36 | 4 | 9 | -1.71 | 134.91 | 0.56 | 0 | Accepted |
| 12 | Rutin |  | 610.57 | 10 | 16 | -1.45 | 269.43 | 0.17 | 3 | Rejected |
| 13 | Isovitexin |  | 432.41 | 7 | 10 | -0.06 | 181.05 | 0.55 | 1 | Accepted |
| 14 | Apigenin-8-C-glucoside |  | 432.38 | 7 | 10 | -2.02 | 181.05 | 0.55 | 1 | Accepted |
| 15 | Hyperoside |  | 464.41 | 8 | 12 | -0.59 | 210.51 | 0.17 | 2 | Rejected |
| 16 | Verbenalin |  | 388.41 | 4 | 10 | -2.26 | 151.98 | 0.11 | 0 | Accepted |
| 17 | kaempferol 7-neohesperidoside |  | 594.57 | 9 | 15 | -0.99 | 249.20 | 0.17 | 3 | Rejected |
| 18 | 4-Methoxysalicylic acid |  | 168.15 | 2 | 4 | 0.74 | 66.76 | 0.85 | 0 | Accepted |
| 19 | Luteolin-4'-O-glucoside |  | 448.38 | 7 | 11 | -2.10 | 190.28 | 0.17 | 2 | Rejected |
| 20 | Isoorientin |  | 448.41 | 8 | 11 | -0.32 | 201.28 | 0.17 | 2 | Rejected |
| 21 | 3-hydroxy-4-methoxyxanthen-9-one |  | 242.23 | 1 | 4 | 1.13 | 59.67 | 0.55 | 0 | Accepted |
| 22 | 3,4',5-Trihydroxy-7-methoxyflavanone |  | 302.28 | 3 | 6 | 0.15 | 96.22 | 0.55 | 0 | Accepted |
| 23 | Iridin |  | 522.50 | 6 | 13 | 0.11 | 197.74 | 0.17 | 3 | Rejected |
| 24 | Amarogentin |  | 576.55 | 7 | 14 | 0.01 | 221.90 | 0.11 | 3 | Rejected |
| 25 | Dihydrokaempferol |  | 288.25 | 4 | 6 | -0.10 | 107.22 | 0.55 | 0 | Accepted |
| 26 | Syringaresinol |  | 418.48 | 2 | 8 | 2.10 | 95.84 | 0.55 | 0 | Accepted |
| 27 | 2,3',4,6-Tetrahydroxybenzophenone |  | 246.22 | 4 | 5 | 0.58 | 97.99 | 0.55 | 0 | Accepted |
| 28 | Luteolin |  | 286.25 | 4 | 6 | 2.07 | 111.13 | 0.55 | 0 | Accepted |
|  | Kaempferide |  | 300.28 | 3 | 6 | 2.02 | 100.13 | 0.55 | 0 | Accepted |
| 29 | Bellidifolin |  | 274.23 | 3 | 6 | 0.02 | 100.13 | 0.55 | 0 | Accepted |
| 30 | Tetrahydroxyxanthone |  | 260.21 | 4 | 6 | 1.90 | 111.13 | 0.55 | 0 | Accepted |
| 31 | (-)-Fustin |  | 288.25 | 4 | 6 | -0.10 | 107.22 | 0.55 |  | Accepted |
| 32 | Trifolirhizin |  | 446.44 | 4 | 10 | 0.54 | 136.30 | 0.55 | 0 | Accepted |
| 33 | Oleanonic acid |  | 454.76 | 1 | 3 | 6.38 | 54.37 | 0.85 | 1 | Accepted |
| 34 | Eriodictyol |  | 288.27 | 4 | 6 | 2.03 | 107.22 | 0.55 | 0 | Accepted |
| 35 | Maslinic acid |  | 472.78 | 3 | 4 | 5.46 | 77.76 | 0.56 | 1 | Accepted |
| 36 | Kaempferol |  | 286.25 | 4 | 6 | 1.77 | 111.13 | 0.55 | 0 | Accepted |
| 37 | Oleanolic acid |  | 456.78 | 2 | 3 | 6.42 | 57.53 | 0.85 | 1 | Accepted |
| 38 | Genistein |  | 270.25 | 3 | 5 | 2.07 | 90.90 | 0.55 | 0 | Accepted |
| 39 | 3-Epilupeol |  | 426.80 | 1 | 1 | 7.40 | 20.23 | 0.55 | 1 | Accepted |
| 40 | Betulinic acid |  | 456.78 | 2 | 3 | 6.52 | 57.53 | 0.85 | 1 | Accepted |
| 41 | Ursolic acid |  | 456.78 | 2 | 3 | 6.47 | 57.53 | 0.85 | 1 | Accepted |

MW: molecule weight; Hdon: hydrogen bond donors; Hacc: hydrogen bond acceptors; Rbon: rotatable bonds; TPSA: topological polar;
